# Experimental study on the bearing capacity and effective anchorage lengths of inclined steel grouting pipes in loess embankment slope

**DOI:** 10.1371/journal.pone.0316528

**Published:** 2024-12-31

**Authors:** Jiawei Fan, Yufang Zhang, Kun Yuan, Wenjiao Zhou

**Affiliations:** 1 Railway Engineering Research Institute, China Academy of Railway Sciences Co. Ltd, Beijing, China; 2 National Key Laboratory of High-Speed Railway Track System, China Academy of Railway Sciences Co. Ltd, Beijing, China; China University of Mining and Technology, CHINA

## Abstract

Splitting grouting is widely used to reinforce unfavorable soil stratum. Inclined steel grouting pipe is a type of structure which can achieve splitting grouting in soil stratum. It has been successfully utilized in argillaceous sandstone stratum, but its application in loess stratum has rarely been studied directly. This research aims to compare and analyze the bearing capacity and effective anchorage lengths of inclined steel grouting pipes with anchorage lengths of 6 m, 9 m, and 12 m. Firstly, bearing capacity of inclined steel grouting pipe was compared with that of ordinary grouting pipe. Secondly, bearing capacity of inclined steel grouting pipe with anchorage lengths of 6 m, 9 m, and 12 m were compared and analyzed. Thirdly, effective anchorage lengths of inclined steel grouting pipe with anchorage lengths of 6 m, 9 m, and 12 m were compared and analyzed. Finally, inclined steel grouting pipe average cohesive strength along effective anchorage length section at the interface between cement grouting and soil stratum was compared with that of rock bolt. The field experimental results illustrated that: (1) The bearing capacity of inclined steel grouting pipe with anchorage length of 9 m increases 22.6% compared with that of ordinary grouting pipe. (2) Anchorage length is not a significant influence factor for bearing capacity of inclined steel grouting pipes in loess embankment slope, while anchorage length is a significant influence factor for modulus of load-displacement curves of inclined steel grouting pipes in loess embankment slope. (3) Effective anchorage length of inclined steel grouting pipe in loess embankment slope will be slightly increased when increasing anchorage length, while the ratio of effective anchorage length to total anchorage length will be decreased when increasing anchorage length. (4) Inclined steel grouting pipe average cohesive strength along effective anchorage length section at the interface between cement grouting and soil stratum is at least three times compared with that of rock bolt.

## Introduction

Loess is a type of sandy silt or clayey silt that is widely distributed in twelve provinces of China [1,2]. Due to its characteristics of water sensitivity and collapsibility, its natural structure is easily damaged so that its strength is decreased significantly when exposed to water [3–6]. Plenty of research has been carried out to investigate mechanical characteristics of loess when it is exposed to water in the China Loess Plateau. Zuo et al. (2020) [7] performed research on the structural degradation of natural and reconstituted loess under long-term seepage, claiming that seepage process damaged the mineral bonding within loess material. Yuan et al. (2019) [8] carried out a series of ring shear tests to investigate residual strength of loess. Sun et al. (2019) [9] conducted indoor model tests of loess slope to investigate slope stability under different rainfall modes, stating that excess pore-water pressure in the slope and poor drainage system were the main reasons for the loess landslide. Wang et al. (2019) [10] analyzed rainfall-induced landslide by means of filed investigation, declaring that impact-erosion effect should be considered in the process of kinetic method analysis. Therefore, the research of loess from the scope of micro to macro has acquired achievement to a certain extent.

Based on the mechanism of loess-water interaction, the technology of grouting was widely utilized to reinforce loess embankment since particle bonding of loess can be increased and hence strength of loess can be enhanced after grouting. A series of research has been carried out to investigate the effectiveness of grouting in loess stratum. Zhao et al. (2021) [11] performed field permeation grouting tests to propose permeation grouting rules in loess stratum, and the method for migration radius prediction were put forward. An et al. (2021) [12] focused on the grouting reinforcement mechanism in loess stratum in Jinan city, proposing dynamic grouting technology based on field loess parameters. Yang et al. (2020) [13] analyzed effects of loess dry density, water content, water to cement ratio, and grouting pressure on uniaxial compression strength and loess strength parameters in Jinan city as well, proposing layered grouting technology. Additionally, jet grouting piles were put into practice to reinforce tunnel foundation of loess stratum, which successfully mitigated settlement deformations [14,15]. Similarly, post-grouting piles were utilized in the field to improve bearing capacity of loess foundation, which enhanced the strength of soil around piles [16–18]. From the early research we can conclude that the study of grouting technology in loess stratum mainly focused on grouting piles. There has been less previous evidence for applications of inclined steel grouting pipes. Inclined steel grouting pipe is an innovative structure which is based on the mechanism of multiple splitting grouting to improve strength parameters of soil stratum, and meanwhile steel pipes could control lateral movements of slopes [19–21]. Therefore, it has the advantages of low construction disturbance, quick construction, high degree of mechanization, and economic performance. The application of inclined steel grouting pipes has been proved to be effective in coal measure stratum and argillaceous sandstone stratum previously [22], but the research of field application of inclined steel grouting pipes in loess stratum has rarely been studied directly. Therefore, it is of great interest to know bearing capacity and effective anchorage lengths of inclined steel grouting pipes in loess stratum.

The objectives of this research are to compare the ultimate bearing capacity of inclined steel grouting pipe and ordinary grouting pipes without splitting grouting, to compare and analyze bearing capacity and effective anchorage lengths of inclined steel grouting pipes with anchorage lengths of 6 m, 9 m, and 12 m in loess embankment slope. To better memorize the specific meanings of these parameters and enhance the readability, definitions of these variables are organized in Table 1.

**Table 1:** Definitions of variables.

Variable	Definition
Bearing capacity	The ultimate load that steel pipe can withstand before failure
Anchorage section	The section that is bonded with surrounding grouting
Free section	The section that is not bonded with surrounding grouting
Effective anchorage length	The distance that loads could be transferred along anchorage section

To thoroughly investigate the bearing capacity and effective anchorage lengths of inclined steel grouting pipe when it is utilized to reinforce loess embankment slope, a series of pull-out tests of inclined steel grouting pipes with different anchorage lengths were conducted at the study site of loess embankment slope. Firstly, to verify the effectiveness of splitting grouting in loess stratum, the ultimate bearing capacity of inclined steel grouting pipe and ordinary grouting pipe without splitting grouting were compared. Additionally, the ultimate bearing capacity of inclined steel grouting pipe with anchorage lengths of 6 m, 9 m, and 12 m were compared. Finally, micro strains at different depths along inclined steel grouting pipes during the process of pull-out tests were measured by installing a series of strain gauges on the outer surface of inclined steel grouting pipes. By analyzing patterns of micro strain at different depths during the process of pull-out tests, effective anchorage lengths of inclined steel grouting pipes in loess stratum were concluded.

This study demonstrates that splitting grouting by inclined steel grouting pipes can successfully and effectively reinforce loess embankment slope, eliminating the traditional doubt that steel grouting pipe cannot provide adequate anchoring force in loess formations due to the collapsibility of loess, and providing a new structure for the reinforcement design of embankment slopes in loess areas.

## Materials and methods

### Study site and materials

**Fig 1:**
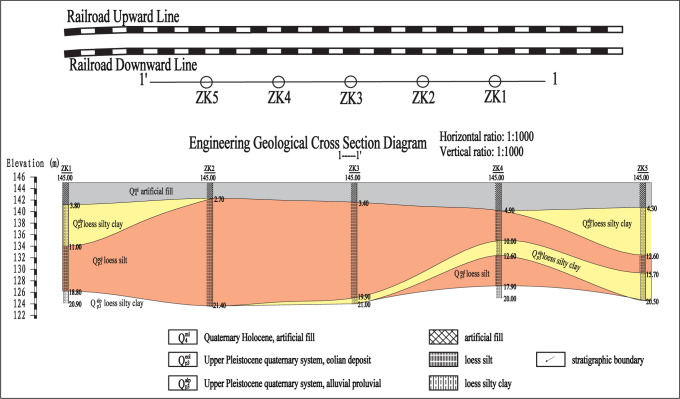
Engineering geological cross section diagram.

**Fig 2:**
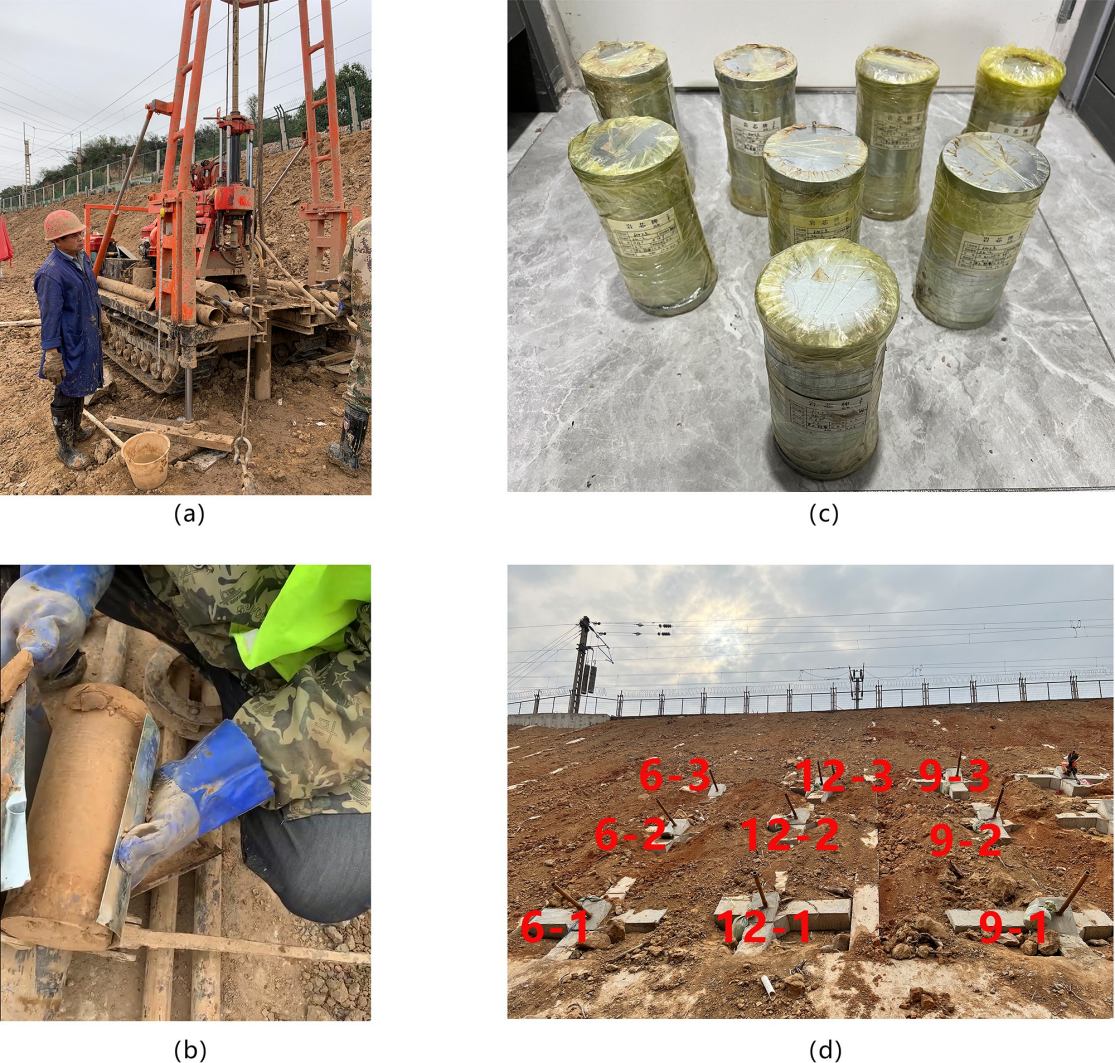
Study site loess properties investigation and overall view of tested inclined steel grouting pipes.

The study site is a loess embankment slope. According to the field geological survey and drilling, the stratum in the tested area is overlying artificial fill and underlying loess silt as well as loess silty clay. The artificial fill layer is about 3~5 m thick, partially mixed with gravel. The underlying loess layer is uniform in texture. The engineering geological cross section diagram is shown in Figs 1 and 2A shows the drilling rig at the survey site. To further investigate engineering properties of loess in the tested area, a series of physical index properties of the loess were tested, including moisture content, particle specific gravity, natural density, dry density, void ratio, saturation, liquid limit, plastic limit, plasticity index, and liquidity index. Fig 2B shows the acquired sample using thin-walled tube sampler. Fig 2C shows packaged soil samples used for physical index properties tests. The physical index properties of the loess are shown in Table 2, and Fig 2D shows inclined steel grouting pipes on loess embankment slope used for pull-out tests.

**Table 2:** The physical index properties of the loess.

Index properties	Unit	Sample number	Average	Maximum	Minimum
Moisture content	%	18	21.96	26.60	17.80
Specific gravity	--	18	2.71	2.73	2.70
Natural density	g/cm^3^	18	1.96	2.03	1.84
Dry density	g/cm^3^	18	1.61	1.72	1.52
Void ratio	--	18	0.68	0.78	0.57
Saturation	%	18	86.83	96.00	73.00
Liquid limit	%	18	28.03	36.20	25.30
Plastic limit	%	18	18.58	21.60	17.30
Plasticity index	--	18	9.46	14.60	8.00
Liquidity index	--	16	0.42	0.74	0.10

### Authorized permits

The experimental research was conducted under the permits of China Railway Zhengzhou Group Co. Ltd. which has the authority of operation and maintenance of the study site.

### Testing equipment

**Fig 3:**
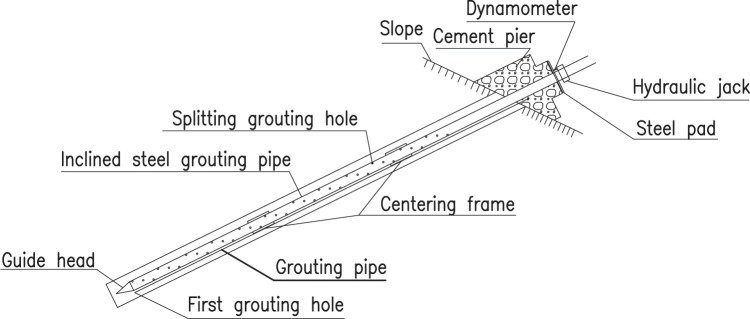
Theoretical structure diagram of testing equipment.

**Fig 4:**
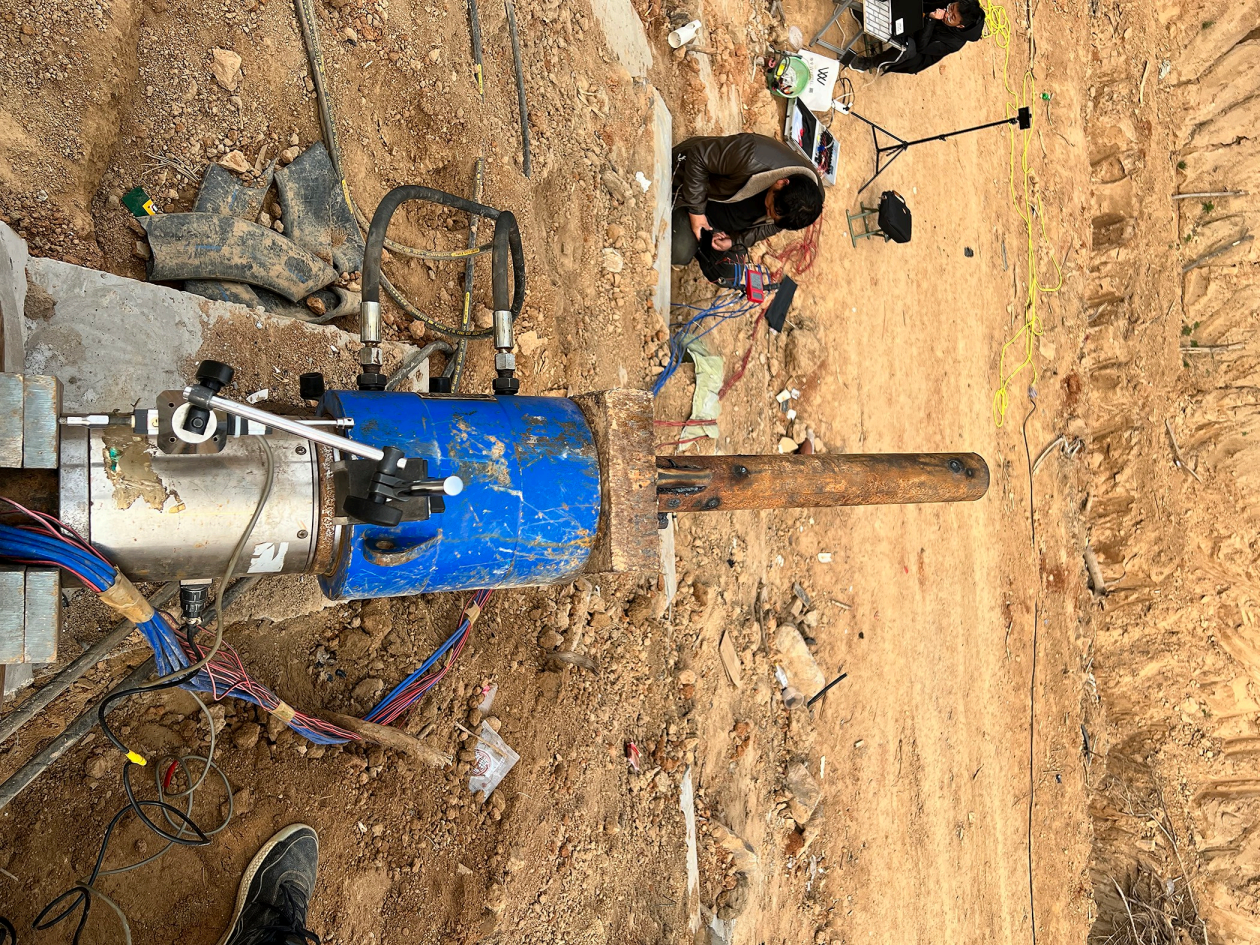
Realistic structure diagram of testing equipment.

To conduct pull-out tests of inclined steel grouting pipes, the following equipment and engineering structures are prepared in advance, including steel pipes grouted in the stratum, cement piers, a steel pad, a vibrating string dynamometer, and a hydraulic jack with measurement capacity of 600 kN. The theoretical and realistic structure diagram of testing equipment are shown in Fig 3s and 4.

**Fig 5:**
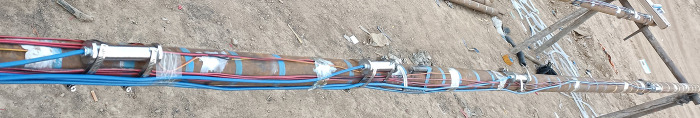
The equipped sensors on steel pipes.

Steel grouting pipes adopt ϕ60 mm × 5 mm seamless steel. To measure strains along steel grouting pipes in the process of pull-out tests, a series of strain gauges are equipped on the outer surface of inclined steel grouting pipes. The equipped sensors on steel pipes are shown in Fig 5.

As for testing data reading and recording equipment, vibrating string dynamometer mentioned above is used for acquiring pull-out forces. A VW-100 vibrating string reading meter is used for reading strain of strain gauges, and an AFT-CM-32 static resistance strain gauge box is used for recording strains. A dial gauge and universal magnetic dial gauge stand are used for reading elongation displacement of inclined steel grouting pipes during pull-out tests.

The unique grouting technology is of great significance to construct inclined steel grouting pipes grouted in the stratum. According to the previous experimental grouting design tests conducted on the study site [23], the slurry with water-cement ratio of 0.5 is used for the first grouting. The method of inverted grouting from the bottom of the hole to the top of grouting pipe is used to fill the gap between the drillhole and steel pipe. The secondary spitting grouting is carried out 6 ~ 12 hours after the first grouting. The slurry with water-cement ratio of 0.7 is used for spitting grouting. The splitting pressure of the grouting is 3 ~ 5 MPa, and the stable pressure is 1.5 ~ 2.5 MPa.

### Testing methods

The test adopts the cyclic loading and unloading method. A hydraulic jack is used to load and unload inclined steel grouting pipes grouted in the stratum. The load is cyclically applied step by step. Inclined steel grouting pipes loading and unloading grade table is shown in Table 3.

**Table 3:** Inclined steel grouting pipes loading and unloading grade table.

Loading and unloading cycles	Load increment: percentage of ultimate tensile strength													
Cycle 1	10							30						10
Cycle 2	10	30						40					30	10
Cycle 3	10	30	40					50				40	30	10
Cycle 4	10	30	40	50				60			50	40	30	10
Cycle 5	10	30	40	50	60			70		60	50	40	30	10
Cycle 6	10	30	40	50	60	70		80	70	60	50	40	30	10
Cycle 7	10	30	40	50	60	70	80	90						
Observation time (min)	5	5	5	5	5	5	5	15	5	5	5	5	5	5

The loading and unloading rates are supposed to decrease correspondingly with the increase of number of cycles. The loading rate of the fifth cycle is controlled within 100 kN/min, and loading rate of the sixth and seventh cycle are controlled within 50 kN/min. During the observation time in each load increment, the number of times of reading the dial gauge is at least 3 times. Within the observation time of each load increment, when the displacement increment does not exceed 0.1 mm, the load of the next level can be applied, otherwise the observation time should be extended. In this case, observe the displacement increment until it is less than 2.0 mm within 2 hours.

When one of the following situations occurs in the test, the test should be stopped in time.

1. Displacement of inclined steel grouting pipe caused by the latter load reaches or exceeds 2 times the displacement caused by the previous load.
2. Displacement of inclined steel grouting pipe continues to increase.
3. Inclined steel grouting pipe is damaged.

### Testing plans

In this experiment, the anchorage lengths of inclined steel grouting pipes are designed to vary by 6 m, 9 m, and 12 m (3 groups each). The reason for selecting such three lengths of anchorage section is based on the needs of realistic application. Since lengths of steel pipes are manufactured as multiple of 3 meters, and 6 meters, 9 meters, and 12 meters are the typical designed anchorage lengths in the realistic design, inclined steel grouting pipes with anchorage lengths of 6 meters, 9 meters, and 12 meters are selected in practical engineering applications. To achieve the control of anchorage lengths, PVC pipes of different lengths are adopted to cover the outer surface of steel pipe where sets as non-grouting free section, namely this non-grouting free section of steel pipe could deform unrestrictedly since this section is not bonded with surrounding grouting. Inclined steel grouting pipes experimental design table is shown in Table 4.

**Table 4:** Inclined steel grouting pipes experimental design table.

Numbering	anchorage lengths	Grouting types	Experiment types	
6–1	6	With splitting grouting	Bearing capacity test	effective anchorage lengths test
6–2	6	With splitting grouting	Bearing capacity test	effective anchorage lengths test
6–3	6	With splitting grouting	Bearing capacity test	effective anchorage lengths test
9–1	9	Without splitting grouting	Bearing capacity test	effective anchorage lengths test
9–2	9	With splitting grouting	Bearing capacity test	effective anchorage lengths test
9–3	9	With splitting grouting	Bearing capacity test	effective anchorage lengths test
12–1	12	With splitting grouting	Bearing capacity test	effective anchorage lengths test
12–2	12	With splitting grouting	Bearing capacity test	effective anchorage lengths test
12–3	12	With splitting grouting	Bearing capacity test	effective anchorage lengths test

## Results

Bearing capacity of inclined steel grouting pipes is a significant parameter since it is critical when conducting slope reinforcement design. In addition, effective anchorage length of inclined steel grouting pipes is also of great importance since it indicates the depth that forces could be transferred to. In this section, firstly, the bearing capacity result from pull-out tests of inclined steel grouting pipe is compared with that of ordinary grouting pipe. Secondly, the bearing capacity results from pull-out tests of inclined steel grouting pipes with different anchorage lengths (6 m, 9 m, and 12 m) are displayed and compared. Thirdly, the effective anchorage lengths results from pull-out tests of inclined steel grouting pipes with different anchorage lengths (6 m, 9 m, and 12 m) are displayed and compared.

### Bearing capacity of inclined steel grouting pipes compared with ordinary grouting pipes without splitting grouting

Ordinary grouting pipes are only capable of performing the first grouting process, which results in the separation of the pipe and the surrounding soil stratum into two distinct systems. In contrast, inclined steel grouting pipe, with subjected to the splitting grouting process, forms a bond with the surrounding soil stratum. The split cement grout fills the clearance between soil particles and provides a skeleton for the loess embankment.

**Fig 6:**
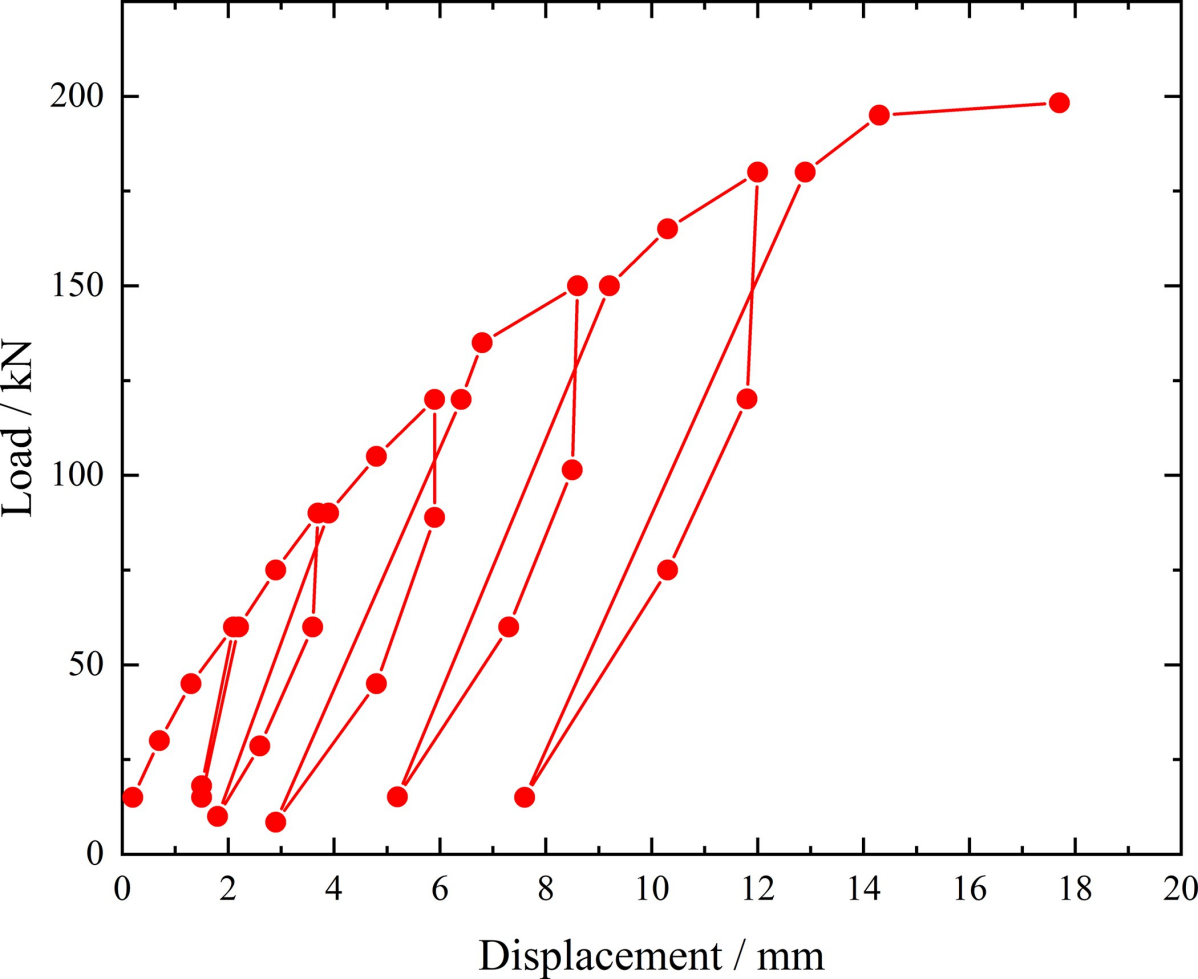
Load-displacement curve during loading and unloading of ordinary grouting pipe of number 9–1.

**Fig 7:**
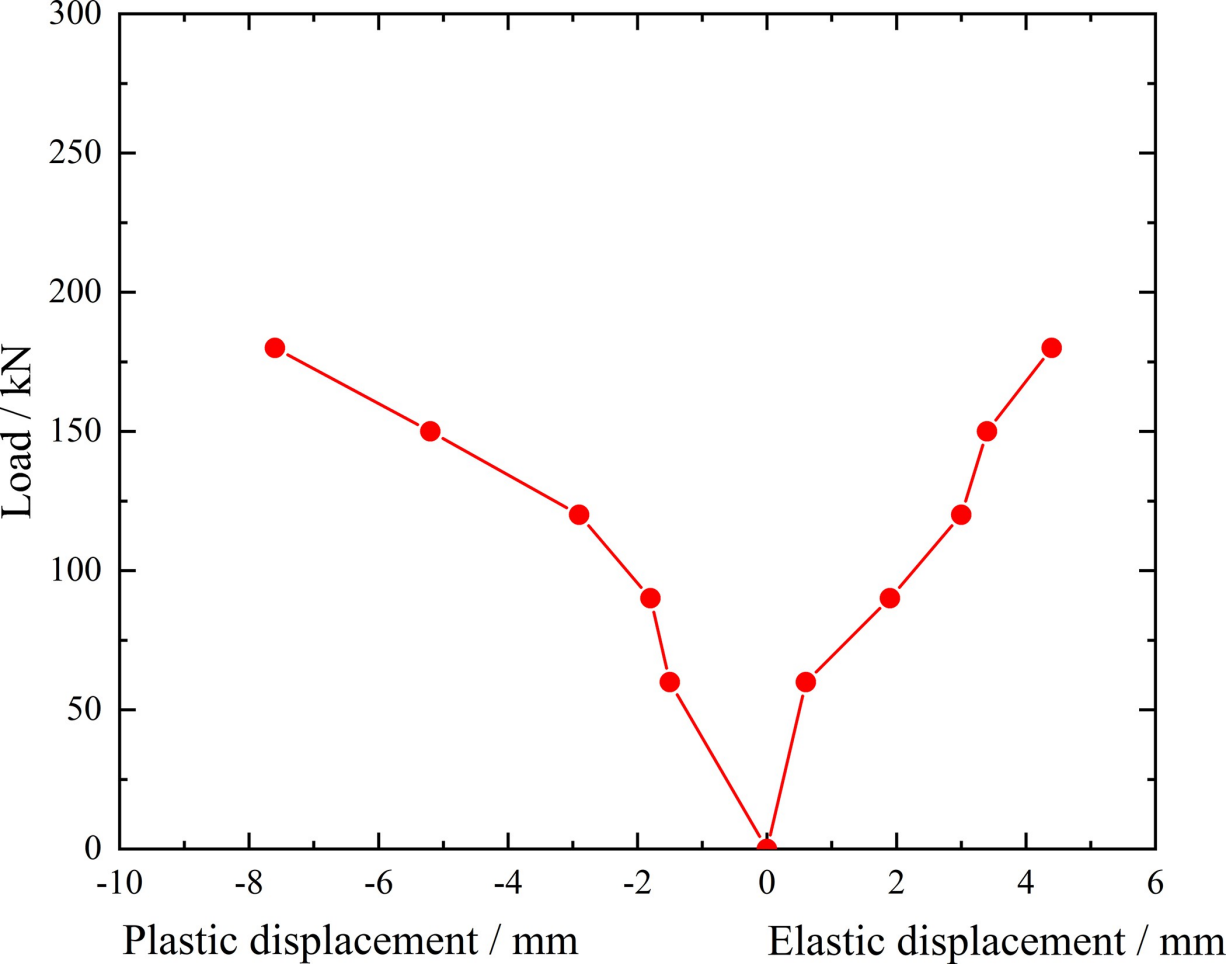
Load-elastic and plastic displacement curve during loading and unloading of ordinary grouting pipe of number 9–1.

**Fig 8:**
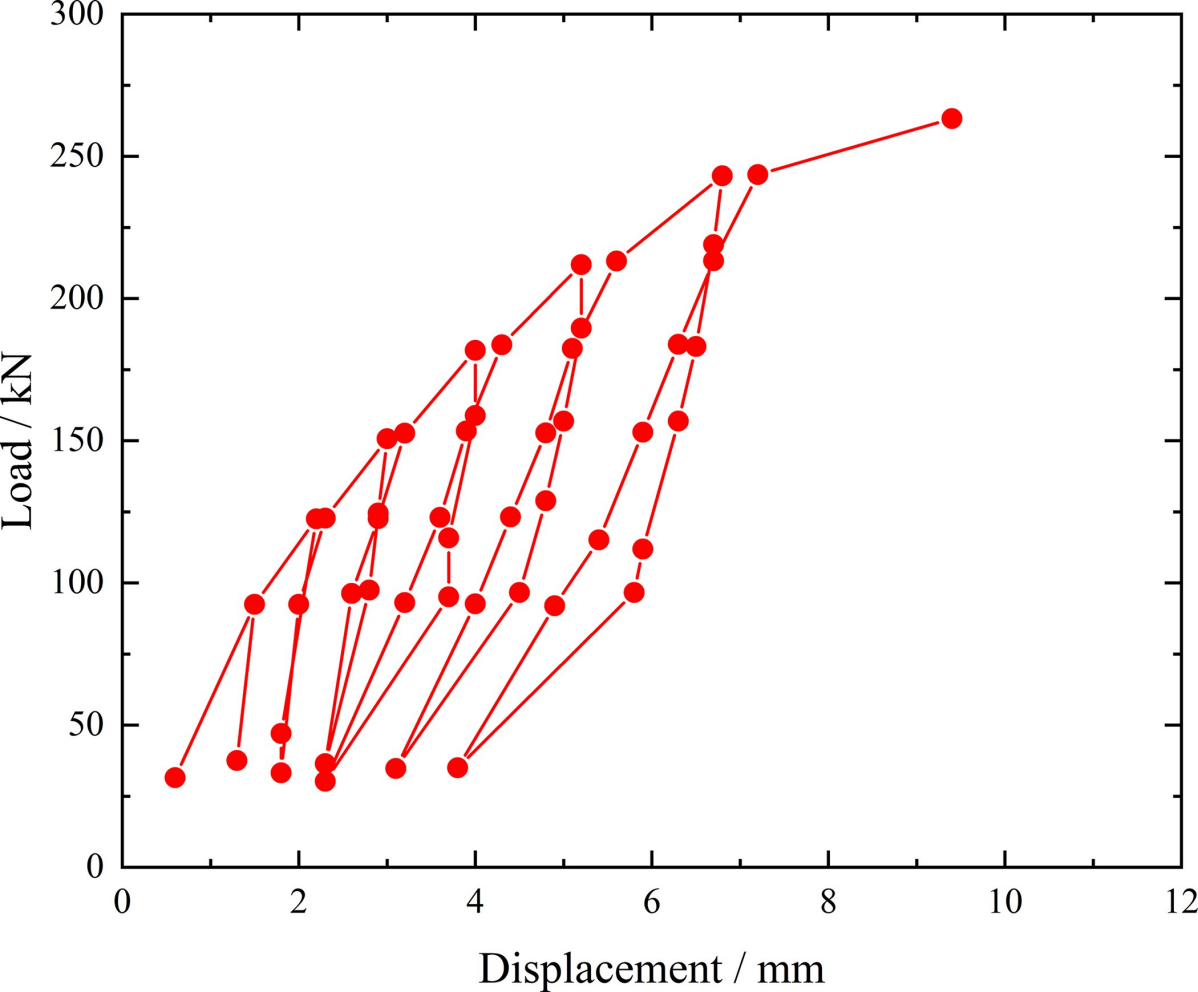
Load-displacement curve during loading and unloading of inclined steel grouting pipe of number 9–2.

**Fig 9:**
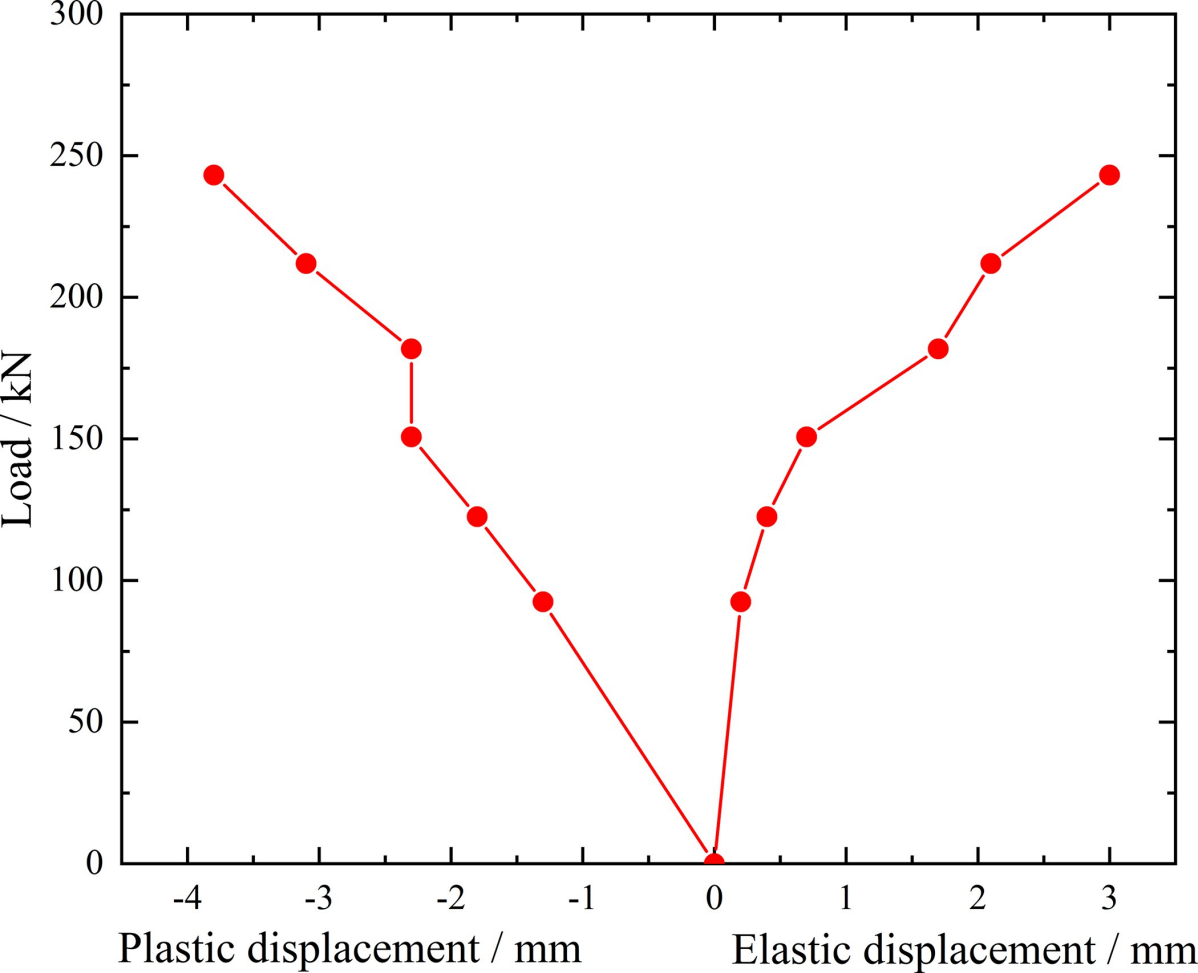
Load-elastic and plastic displacement curve during loading and unloading of inclined steel grouting pipe of number 9–2.

**Fig 10:**
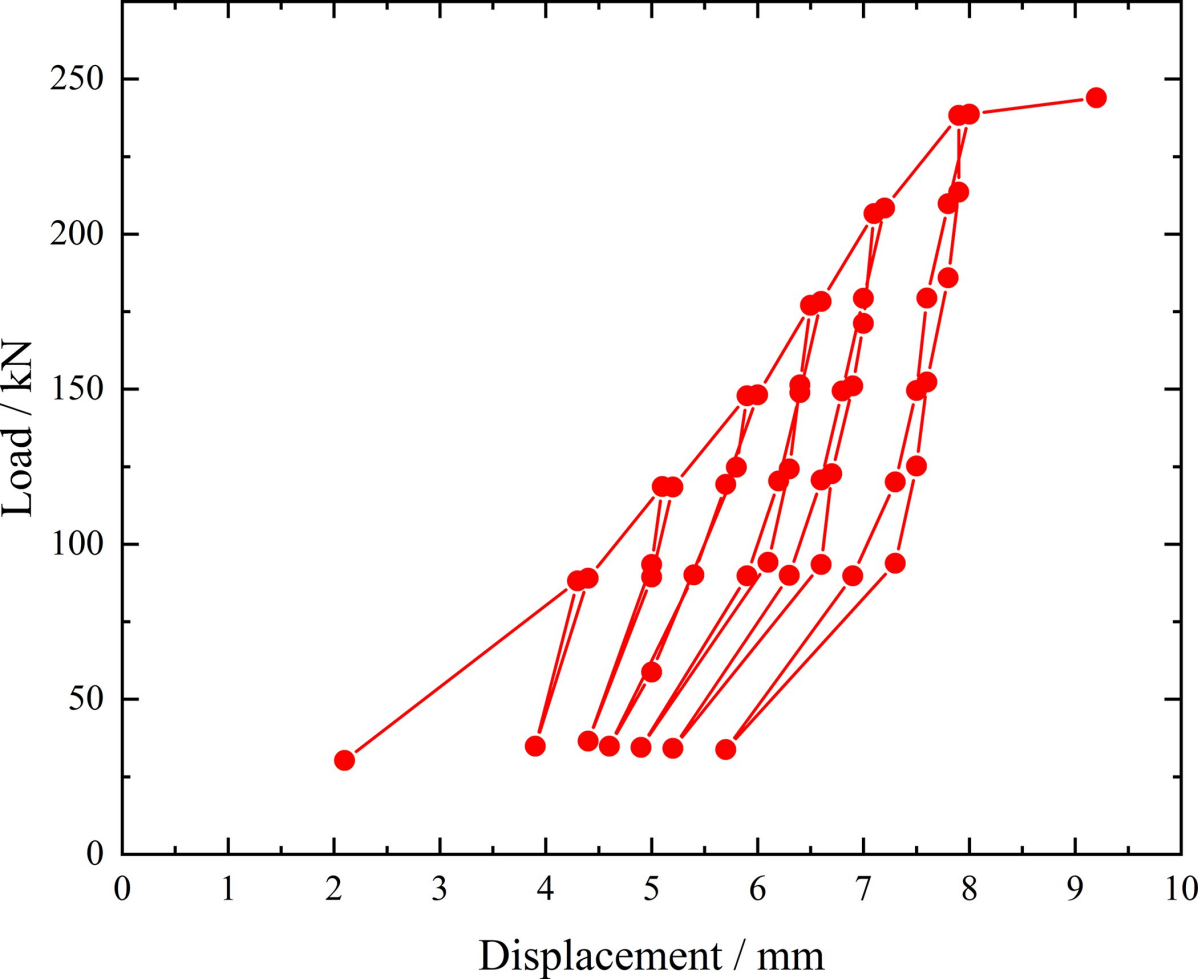
Load-displacement curve during loading and unloading of inclined steel grouting pipe of number 9–3.

**Fig 11:**
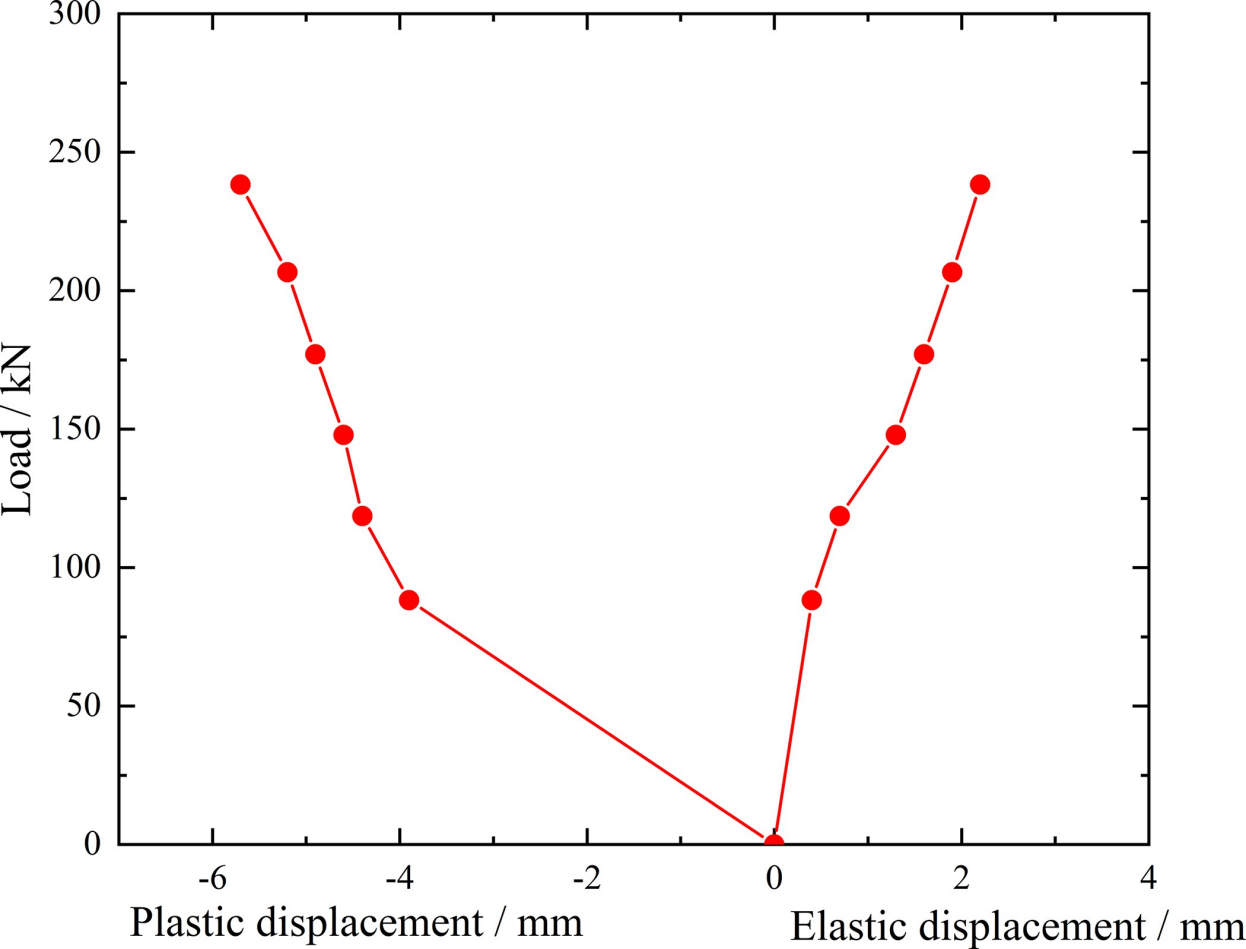
Load-elastic and plastic displacement curve during loading and unloading of inclined steel grouting pipe of number 9–3.

To verify the effect of splitting grouting, the anchorage length of inclined steel grouting pipes should be controlled as a non-changeable factor. In this experiment, an anchorage length of 9 m is selected. As shown in Fig 2D, inclined steel grouting pipes with anchorage length of 9 m are grouted in loess embankment slope. The numbers of them are labeled as 9–1, 9–2, and 9–3. The pipe of number 9–1 is an ordinary grouting pipe without splitting grouting, and pipes of number 9–2 and 9–3 are inclined steel grouting pipes. Pull-out tests loading and unloading curves of pipes labeled as number 9–1, 9–2, and 9–3 are displayed in Figs 6–11.

From the testing results of Figs 6 and 7, the displacement of ordinary grouting pipe labeled as number 9–1 is increased obviously when the load is applied to 195 kN. Displacement caused by the latter load exceeds 2 times the displacement caused by the previous load when the load is applied to 198 kN, which meets the test stop rule (1) mentioned in the testing methods section. Therefore, the bearing capacity of ordinary grouting pipe of number 9–1 is 195 kN.

From the testing results of Figs 8 and 9, the displacement of inclined steel grouting pipe labeled as number 9–2 is increased obviously when the load is applied to 244 kN. Displacement caused by the latter load exceeds 2 times the displacement caused by the previous load when the load is applied to 263 kN, which meets the test stop rule (1) mentioned in the testing methods section. Therefore, the bearing capacity of inclined steel grouting pipe of number 9–2 is 244 kN.

From the testing results of Figs 10 and 11, the displacement of inclined steel grouting pipe labeled as number 9–3 is increased obviously when the load is applied to 239 kN. Displacement caused by the latter load exceeds 2 times the displacement caused by the previous load when the load is applied to 244 kN, which meets the test stop rule (1) mentioned in the testing methods section. Therefore, the bearing capacity of inclined steel grouting pipe of number 9–3 is 239 kN.

Compare the bearing capacity testing results of ordinary grouting pipe with inclined steel grouting pipes. The bearing capacity of ordinary grouting pipe with anchorage length of 9 m is 195 kN, and the bearing capacity of inclined steel grouting pipe with anchorage length of 9 m is 239~244 kN. Under the effect of splitting grouting, bearing capacity of inclined steel grouting pipe with anchorage length of 9 m increases 22.6%.

### Bearing capacity of inclined steel grouting pipes with different anchorage lengths

**Fig 12:**
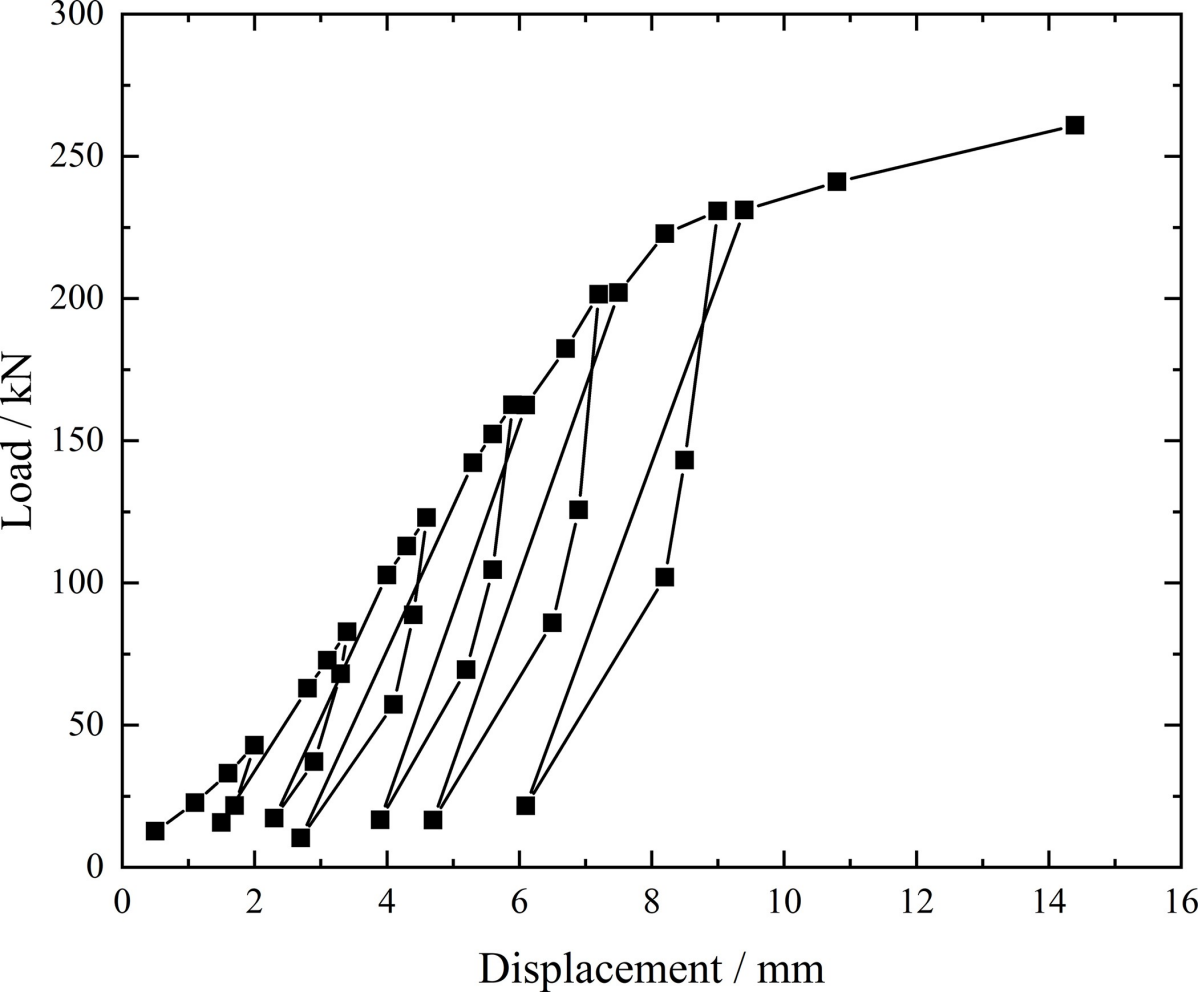
Load-displacement curve during loading and unloading of inclined steel grouting pipe of number 6–1.

**Fig 13:**
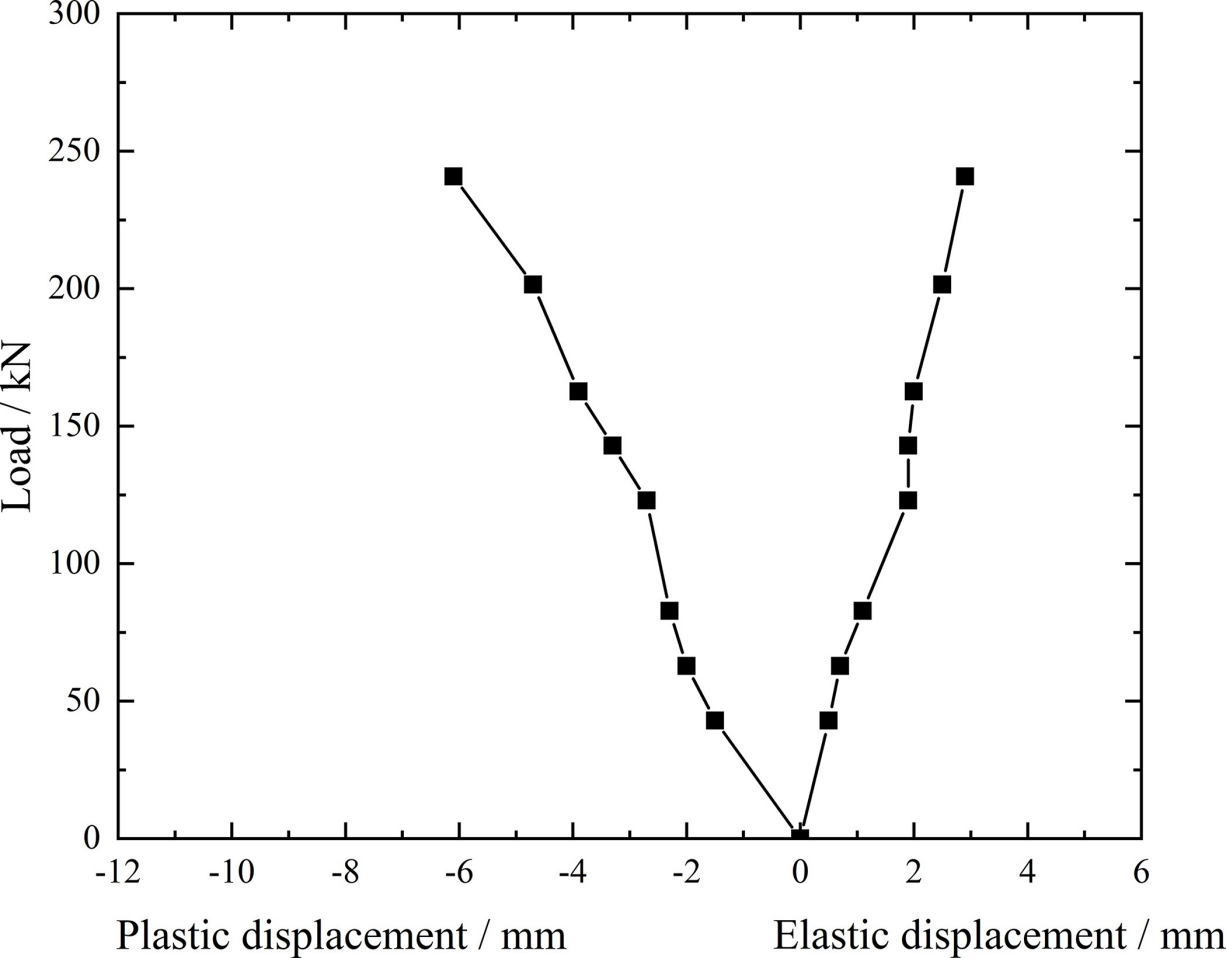
Load-elastic and plastic displacement curve during loading and unloading of inclined steel grouting pipe of number 6–1.

**Fig 14:**
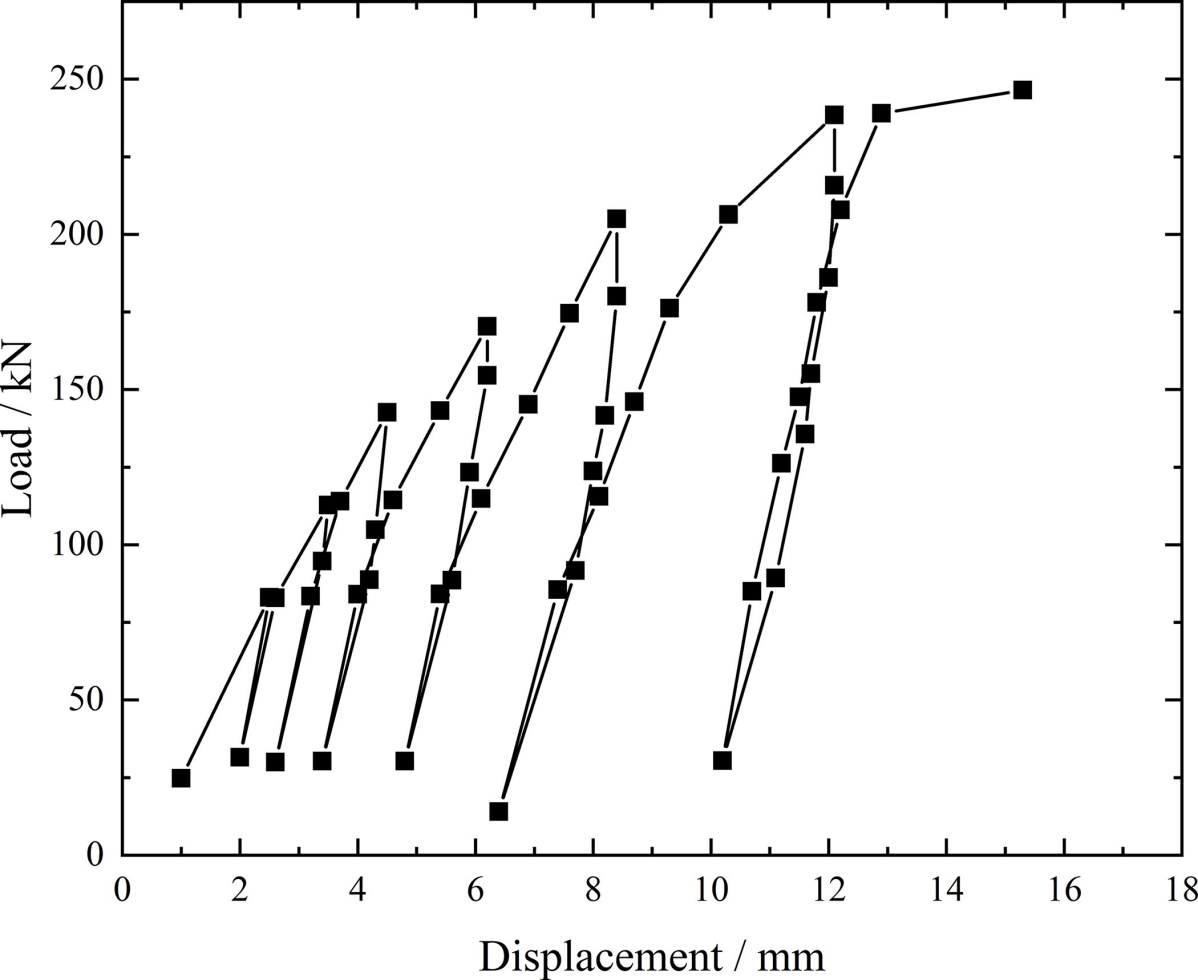
Load-displacement curve during loading and unloading of inclined steel grouting pipe of number 6–2.

**Fig 15:**
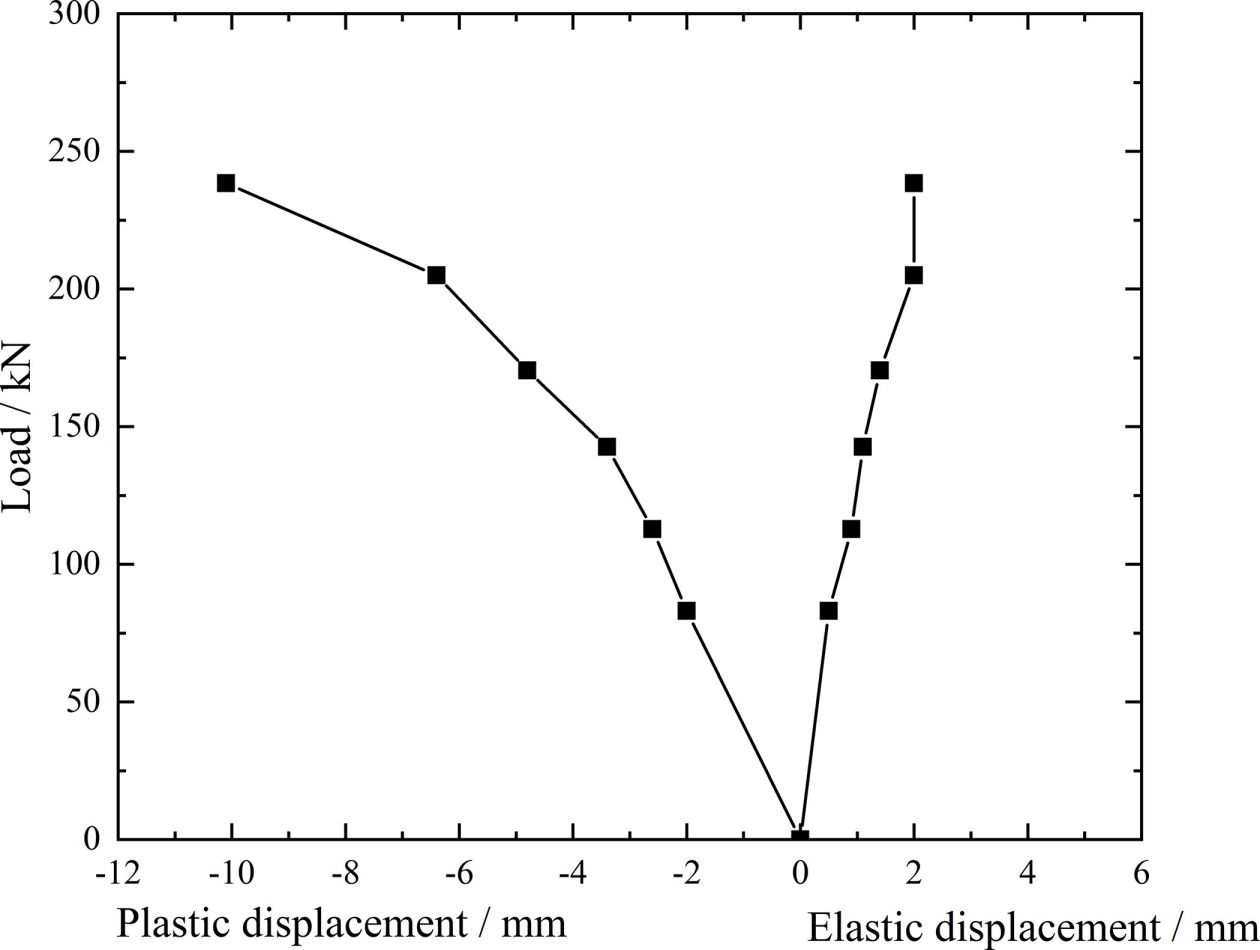
Load-elastic and plastic displacement curve during loading and unloading of inclined steel grouting pipe of number 6–2.

**Fig 16:**
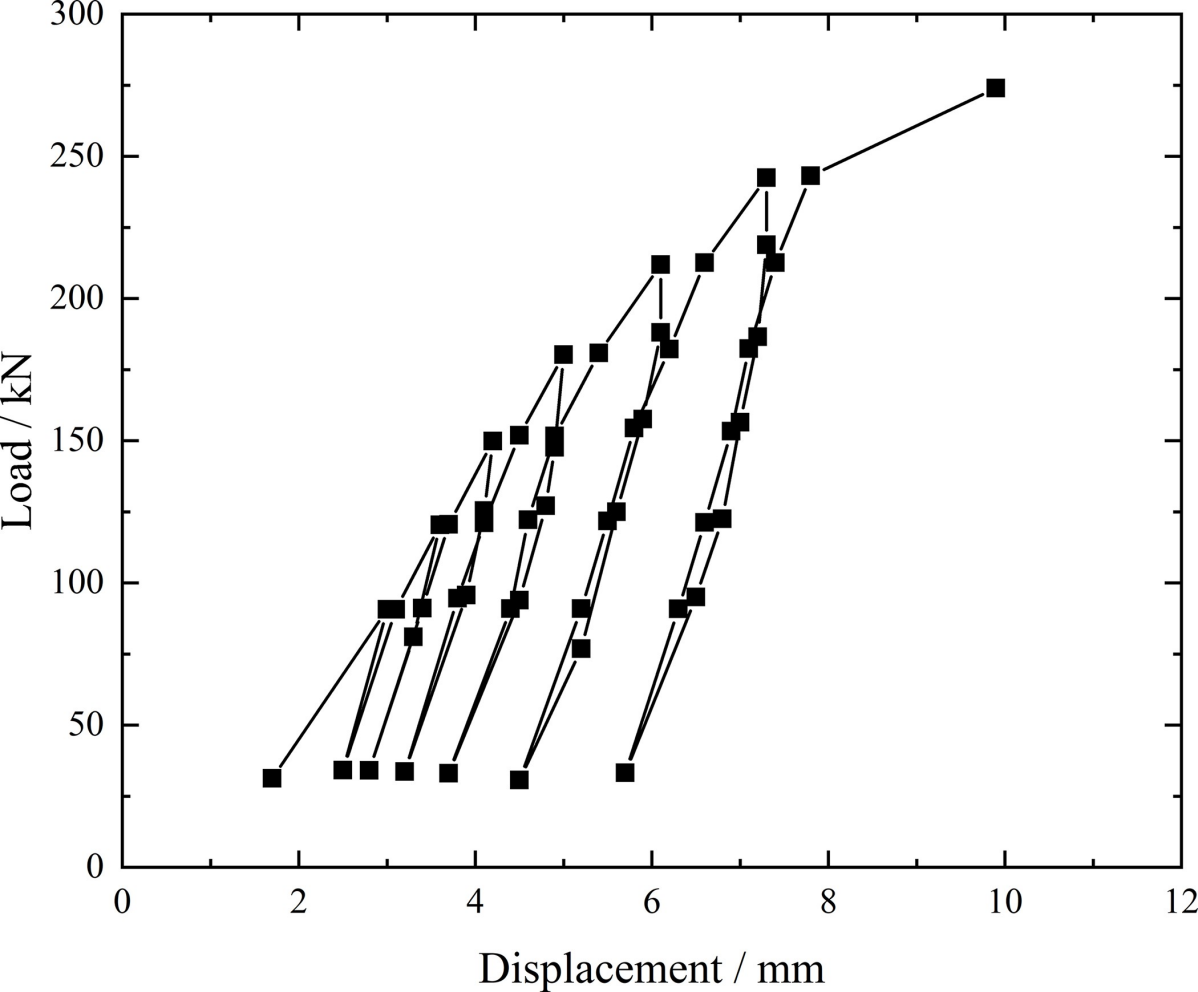
Load-displacement curve during loading and unloading of inclined steel grouting pipe of number 6–3.

**Fig 17:**
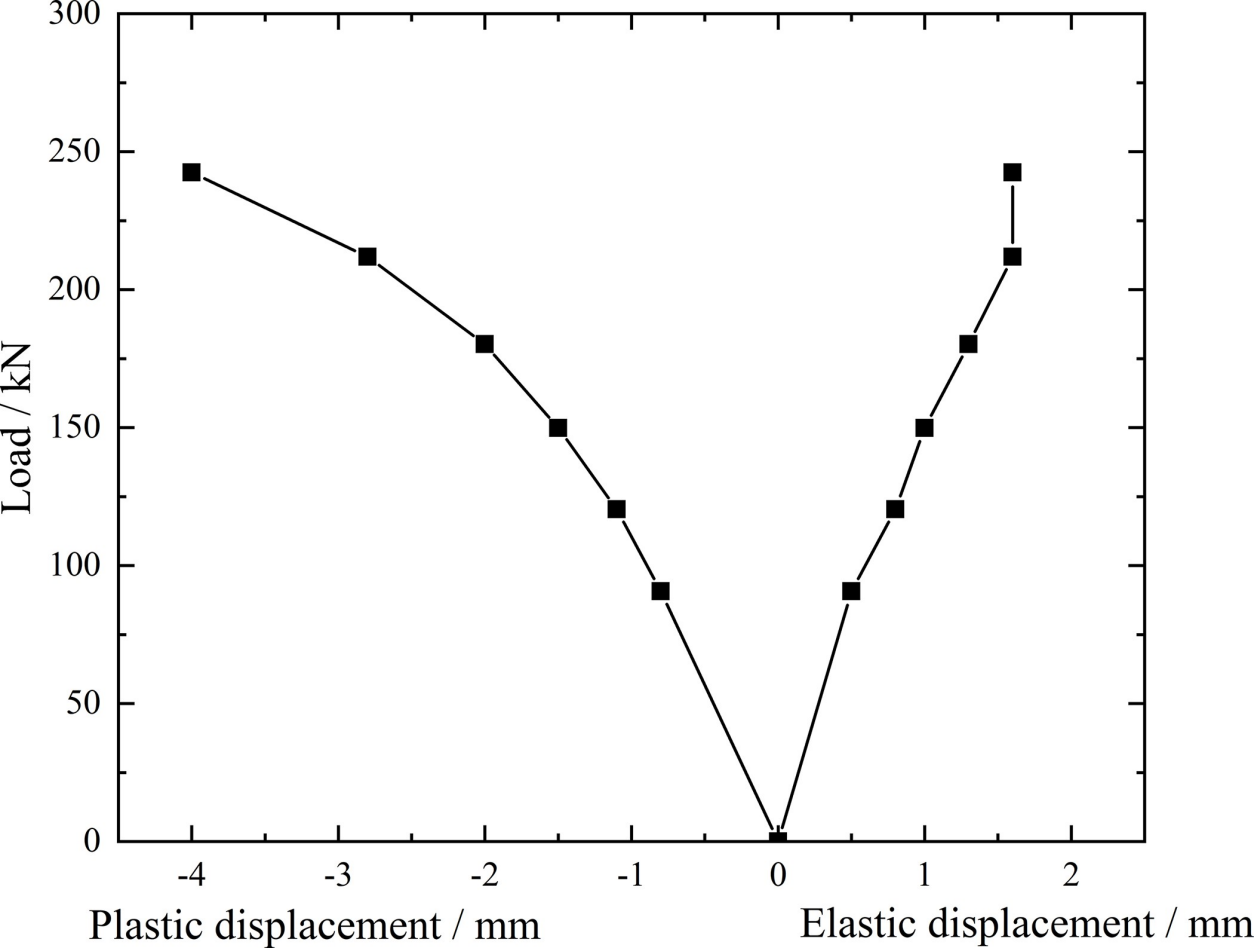
Load-elastic and plastic displacement curve during loading and unloading of inclined steel grouting pipe of number 6–3.

**Fig 18:**
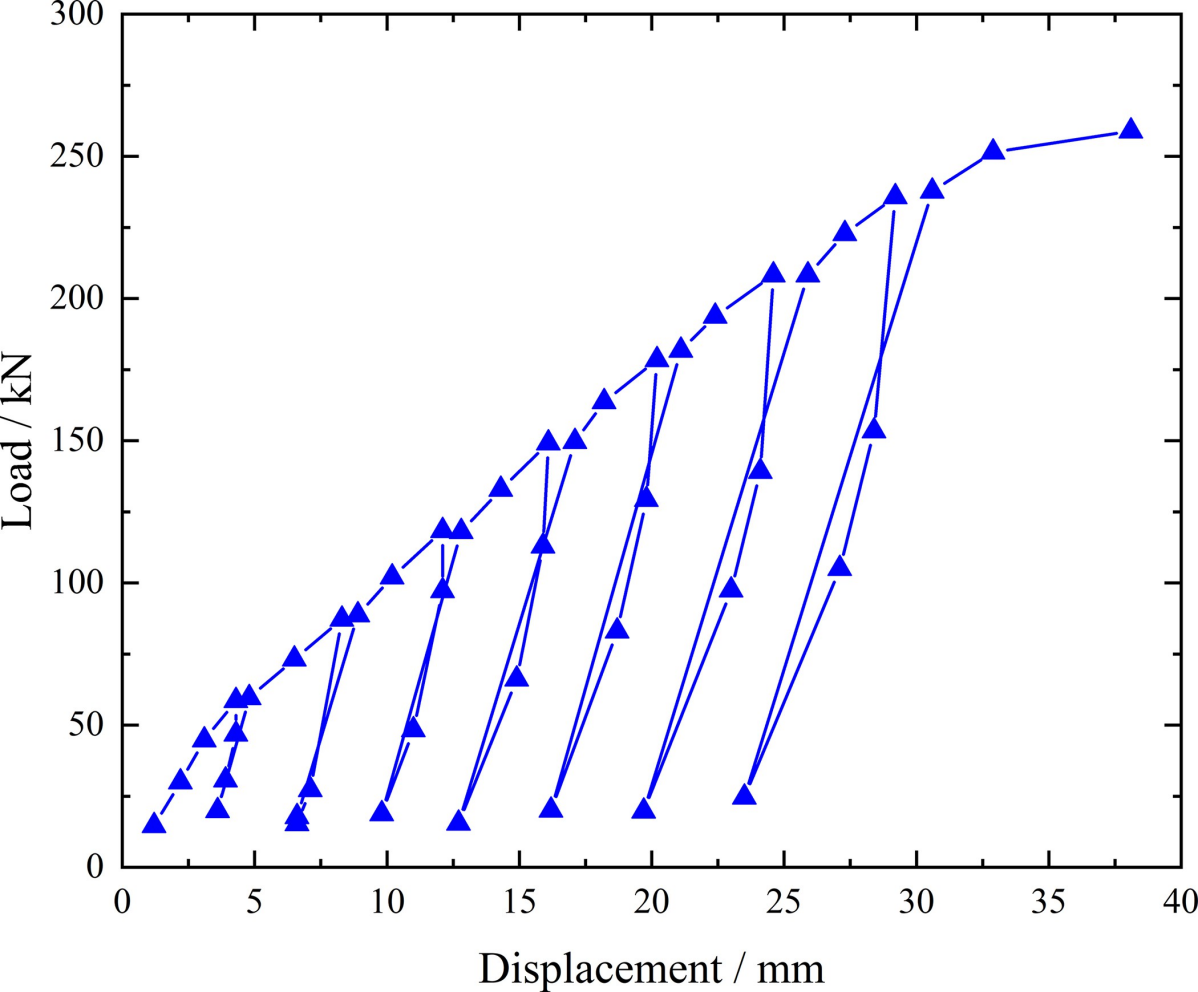
Load-displacement curve during loading and unloading of inclined steel grouting pipe of number 12–1.

**Fig 19:**
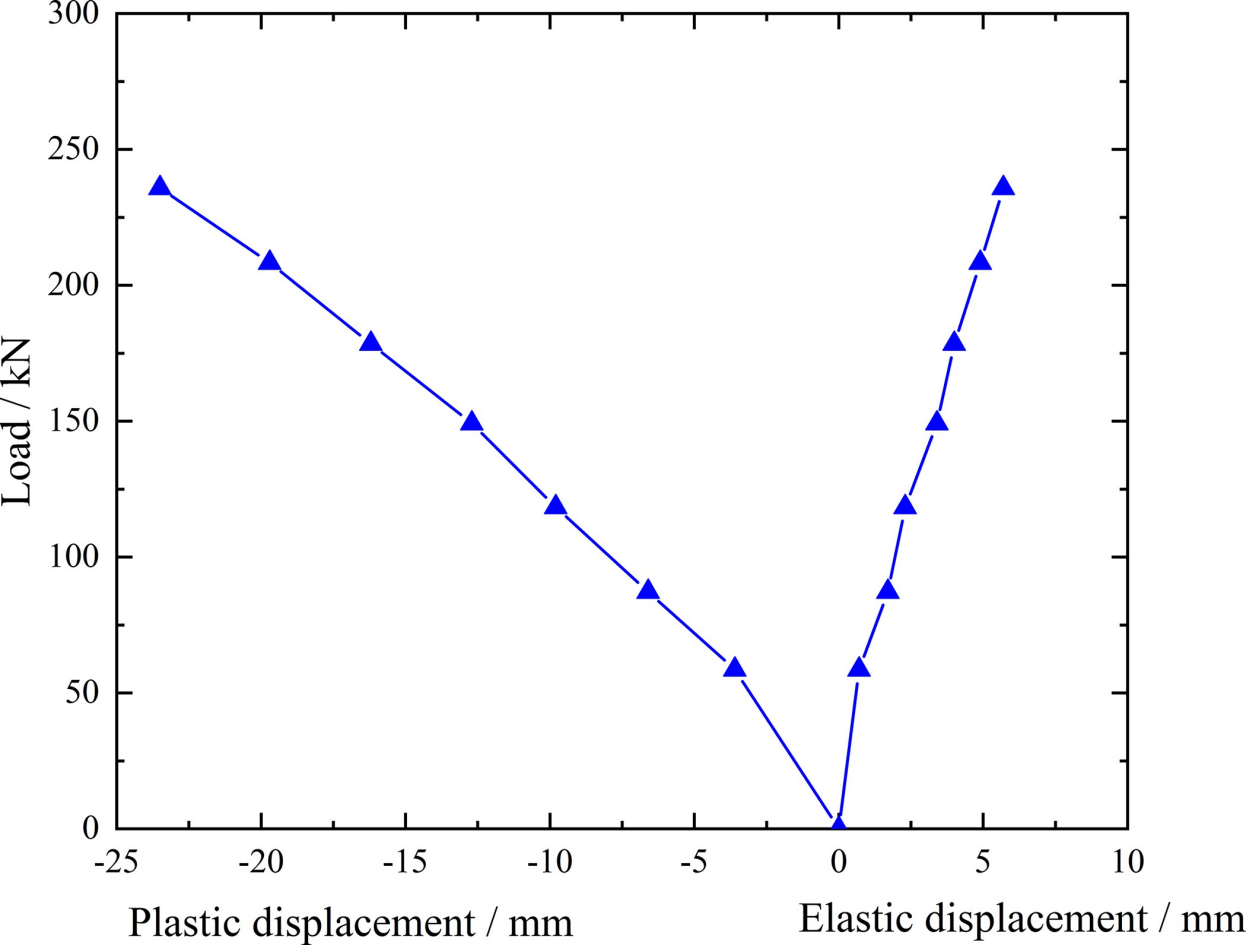
Load-elastic and plastic displacement curve during loading and unloading of inclined steel grouting pipe of number 12–1.

**Fig 20:**
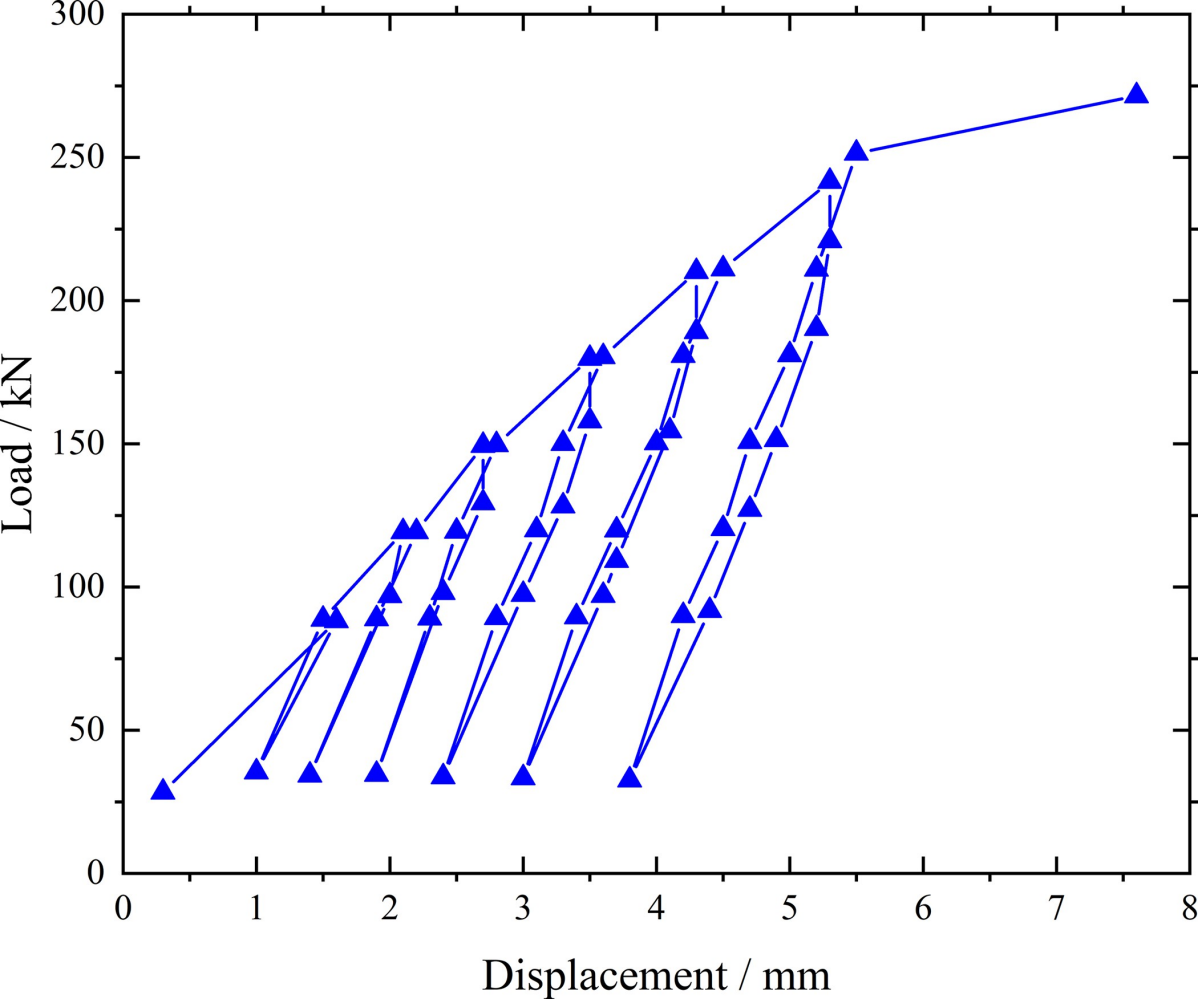
Load-displacement curve during loading and unloading of inclined steel grouting pipe of number 12–2.

**Fig 21:**
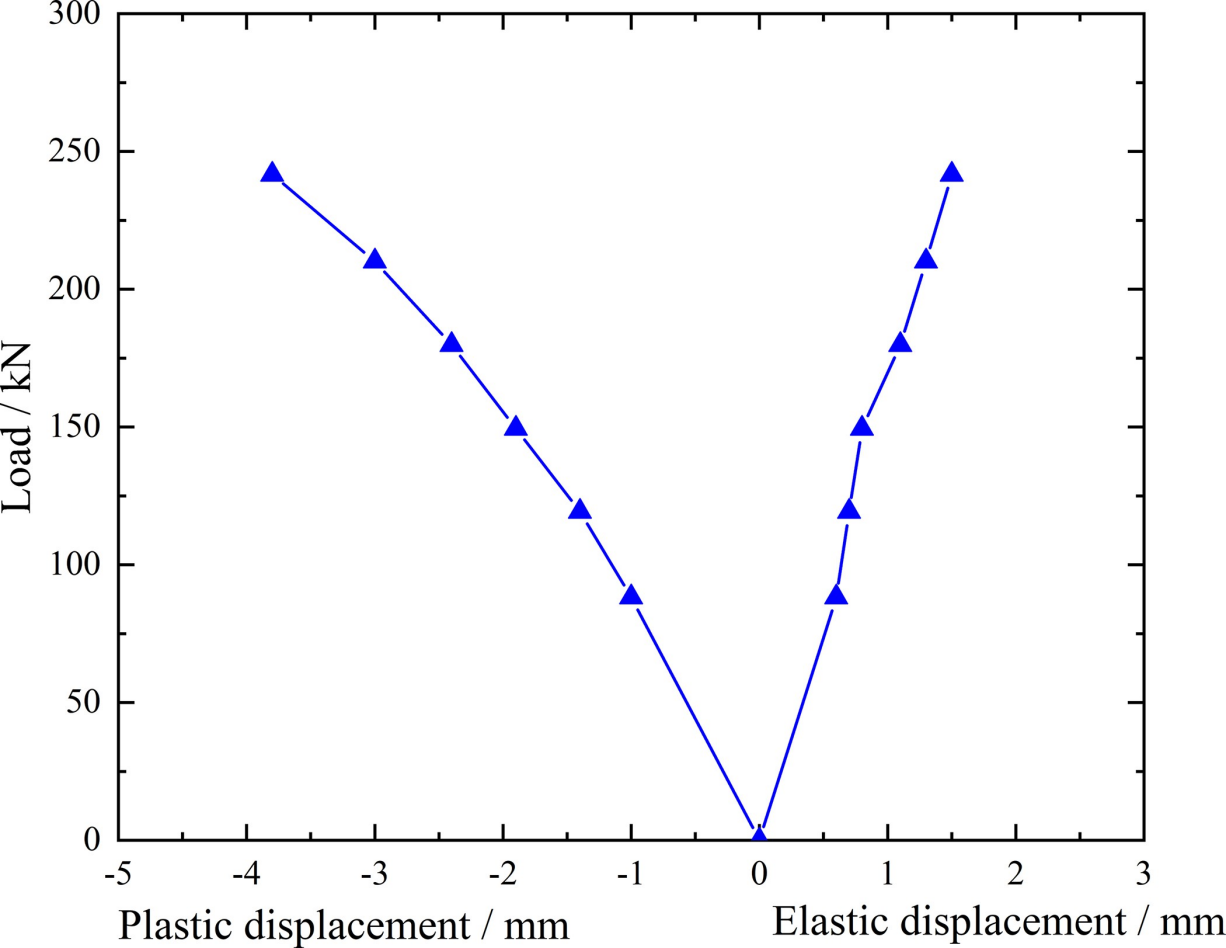
Load-elastic and plastic displacement curve during loading and unloading of inclined steel grouting pipe of number 12–2.

**Fig 22:**
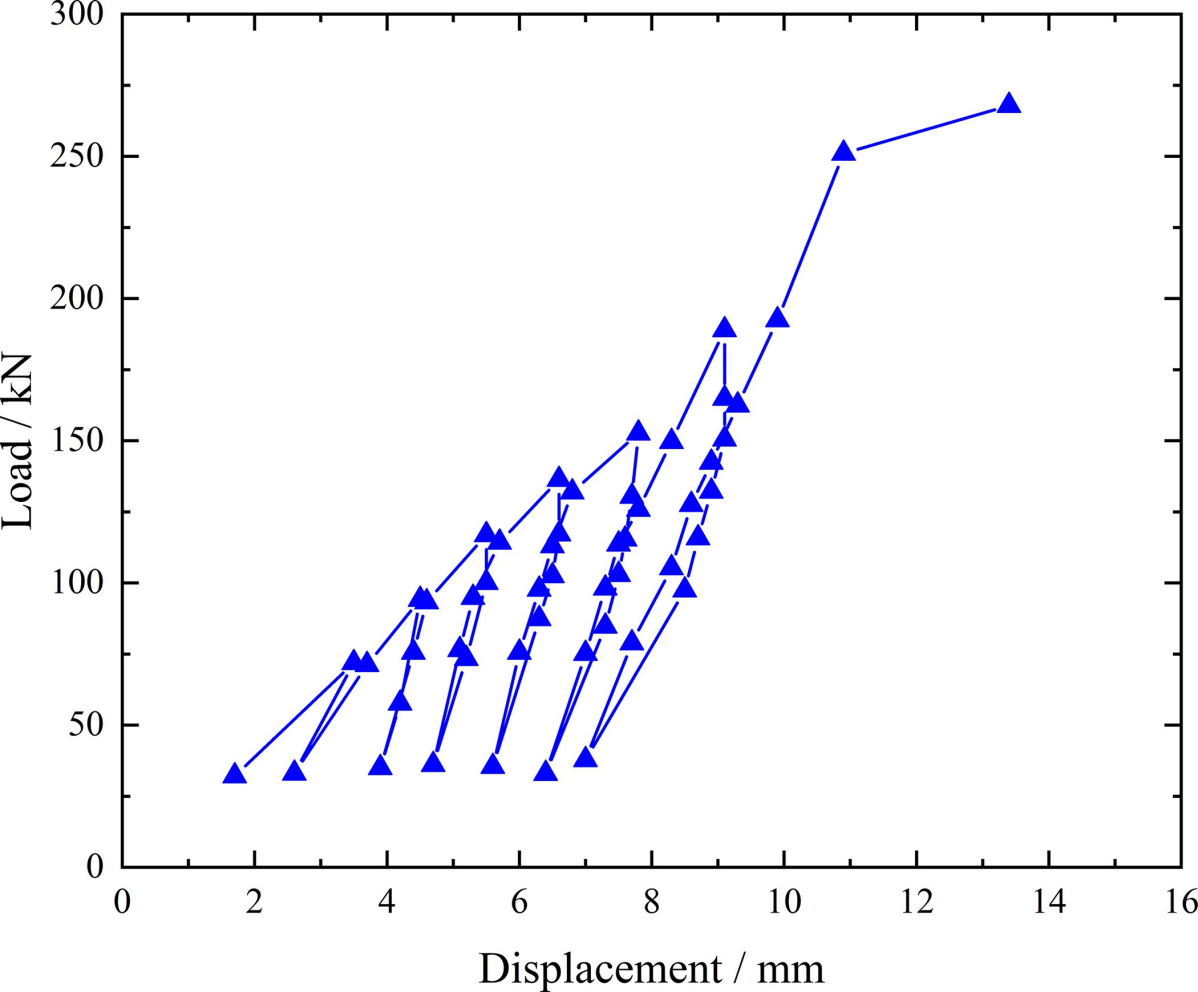
Load-displacement curve during loading and unloading of inclined steel grouting pipe of number 12–3.

**Fig 23:**
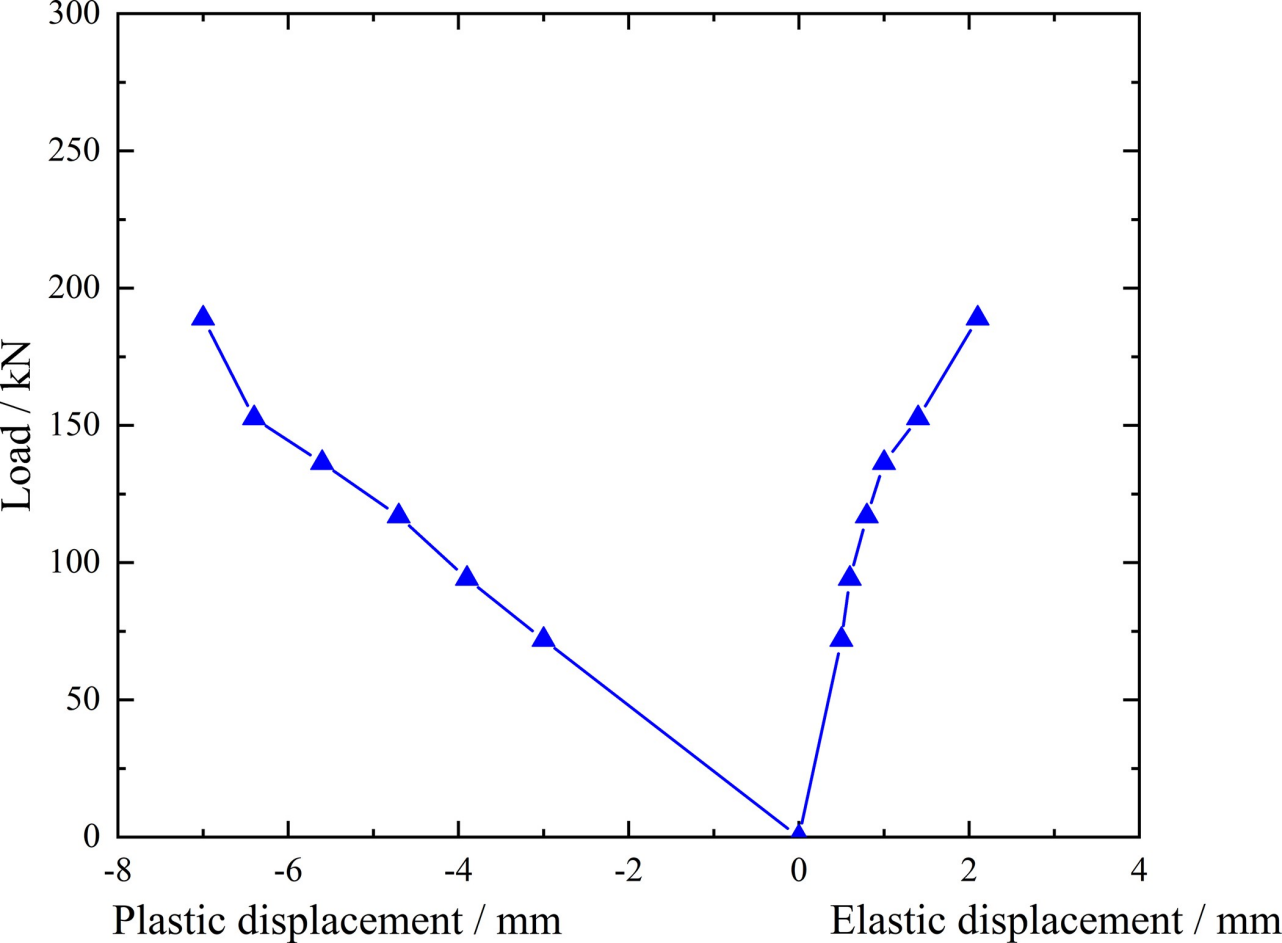
Load-elastic and plastic displacement curve during loading and unloading of inclined steel grouting pipe of number 12–3.

It has been proved that splitting grouting can enhance the bearing capacity of inclined steel grouting pipes to a certain extent. The next step is to compare the bearing capacity of inclined steel grouting pipes with different anchorage lengths. It might be true that the longer the anchorage length is, the higher the bearing capacity will be. Then this supposition will be investigated in this section. Apart from numbers 9–1, 9–2, and 9–3 pipes, as shown in Fig 2D, inclined steel grouting pipes with anchorage length of 6 m and 12 m are grouted in loess embankment slope. The numbers of them are labeled as 6–1, 6–2, 6–3, 12–1, 12–2, and 12–3. Pull-out tests loading and unloading curves of pipes of number 6–1, 6–2, and 6–3 are displayed in Figs 12–17. Pull-out tests loading and unloading curves of pipes of number 12–1, 12–2, and 12–3 are displayed in Figs 18–23.

From the testing results of Figs 12 and 13, the displacement of inclined steel grouting pipe labeled as number 6–1 is increased obviously when the load is applied to 241 kN. Displacement caused by the latter load exceeds 2 times the displacement caused by the previous load when the load is applied to 261 kN, which meets the test stop rule (1) mentioned in the testing methods section. Therefore, the bearing capacity of inclined steel grouting pipe of number 6–1 is 241 kN.

From the testing results of Figs 14 and 15, the displacement of inclined steel grouting pipe labeled as number 6–2 is increased obviously when the load is applied to 239 kN. Displacement caused by the latter load exceeds 2 times the displacement caused by the previous load when the load is applied to 246 kN, which meets the test stop rule (1) mentioned in the testing methods section. Therefore, the bearing capacity of inclined steel grouting pipe of number 6–2 is 239 kN.

From the testing results of Figs 16 and 17, the displacement of inclined steel grouting pipe labeled as number 6–3 is increased obviously when the load is applied to 243 kN. Displacement caused by the latter load exceeds 2 times the displacement caused by the previous load when the load is applied to 274 kN, which meets the test stop rule (1) mentioned in the testing methods section. Therefore, the bearing capacity of inclined steel grouting pipe of number 6–3 is 243kN.

From the testing results of Figs 18 and 19, the displacement of inclined steel grouting pipe labeled as number 12–1 is increased obviously when the load is applied to 251 kN. Displacement caused by the latter load exceeds 2 times the displacement caused by the previous load when the load is applied to 259 kN, which meets the test stop rule (1) mentioned in the testing methods section. Therefore, the bearing capacity of inclined steel grouting pipe of number 12–1 is 251 kN.

From the testing results of Figs 20 and 21, the displacement of inclined steel grouting pipe labeled as number 12–2 is increased obviously when the load is applied to 251 kN. Displacement caused by the latter load exceeds 2 times the displacement caused by the previous load when the load is applied to 271 kN, which meets the test stop rule (1) mentioned in the testing methods section. Therefore, the bearing capacity of inclined steel grouting pipe of number 12–2 is 251 kN.

From the testing results of Figs 22 and 23, the displacement of inclined steel grouting pipe labeled as number 12–3 is increased obviously when the load is applied to 251 kN. At this moment, sixth unloading cycles and seventh loading cycles have been already completed. Therefore, continue to apply load to the inclined steel grouting pipe until large displacement increase occurs. Displacement caused by the latter load exceeds 2 times the displacement caused by the previous load when the load is applied to 268 kN, which meets the test stop rule (1) mentioned in the testing methods section. Therefore, the bearing capacity of inclined steel grouting pipe of number 12–3 is 251 kN.

Compare the bearing capacity testing results of inclined steel grouting pipes with different anchorage lengths. The bearing capacity of inclined steel grouting pipe with anchorage length of 6 m is 239~243 kN, and the bearing capacity of inclined steel grouting pipe with anchorage length of 9 m is 239~244 kN, and the bearing capacity of inclined steel grouting pipe with anchorage length of 12 m is 251 kN. The bearing capacity of inclined steel grouting pipes with anchorage length of 6 m or 9 m are almost at the same level. As for inclined steel grouting pipe with anchorage length of 12 m, its bearing capacity only enhances about 10 kN, which does not make a significant difference. Therefore, it could be concluded that anchorage length is not a significant influence factor for bearing capacity of inclined steel grouting pipes in loess embankment slope.

### Effective anchorage lengths of inclined steel grouting pipes with different anchorage lengths

**Fig 24:**
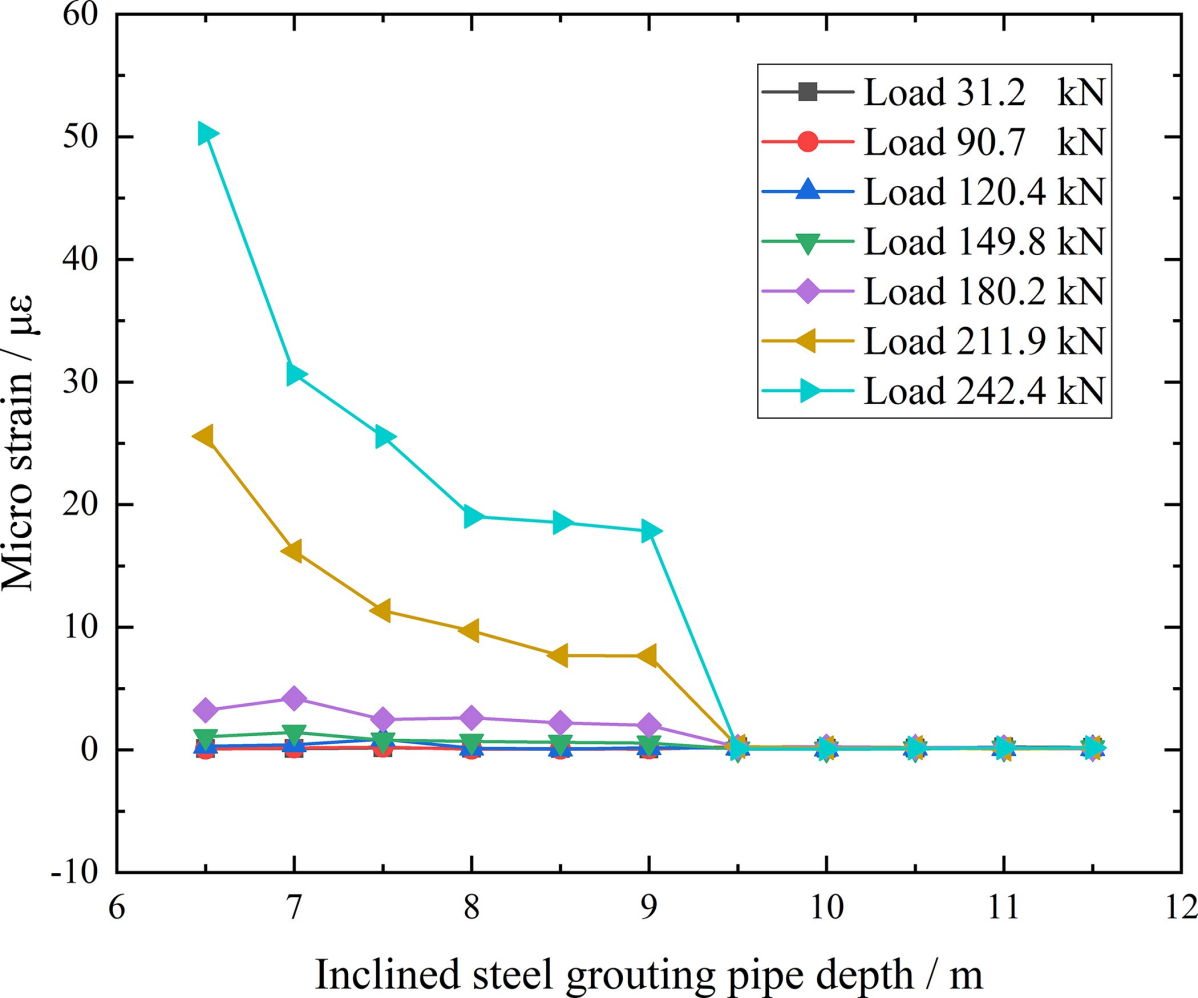
Micro strain corresponding anchorage section depth curve of inclined steel grouting pipe of number 6–3.

**Fig 25:**
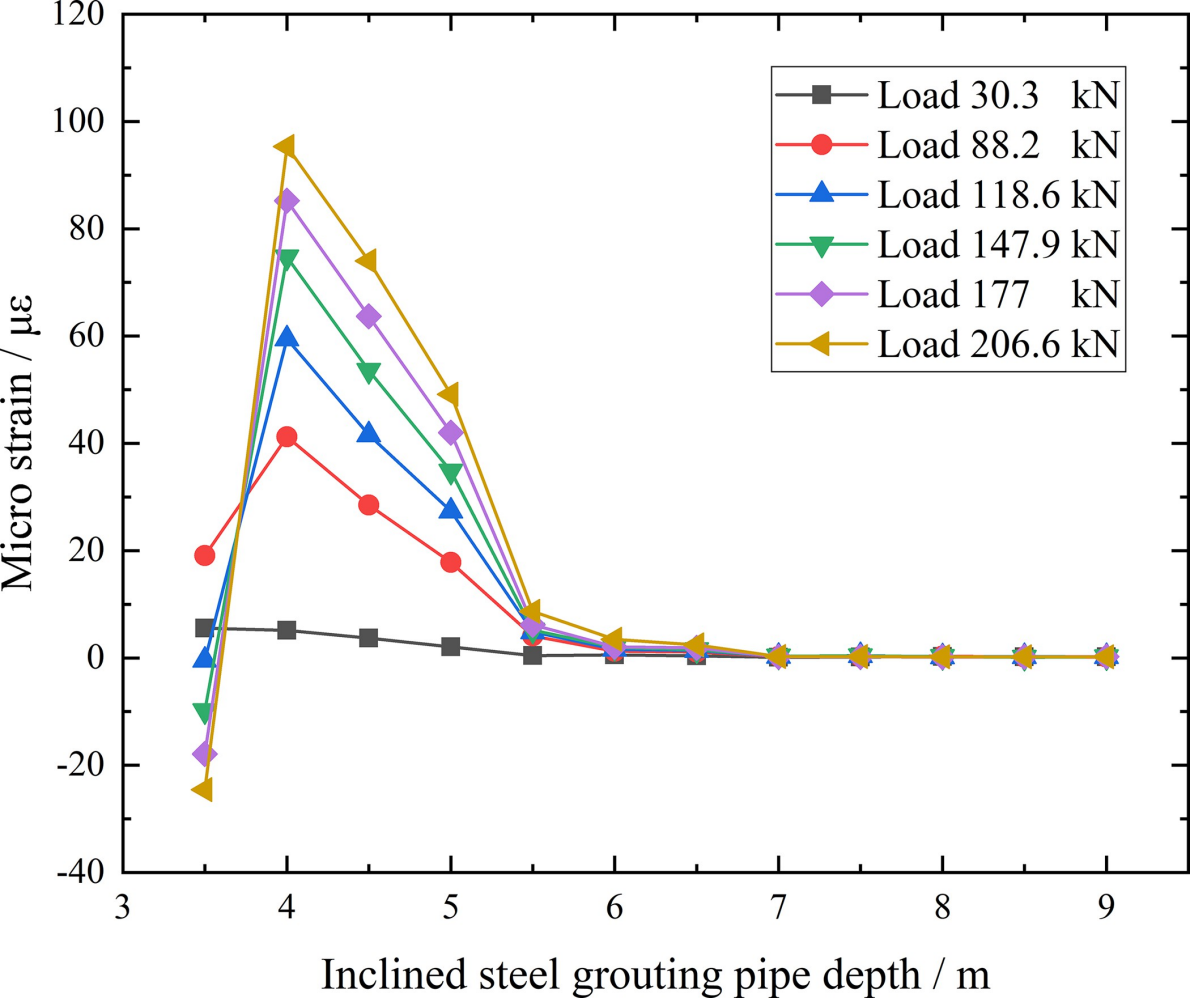
Micro strain corresponding anchorage section depth curve of inclined steel grouting pipe of number 9–3.

**Fig 26:**
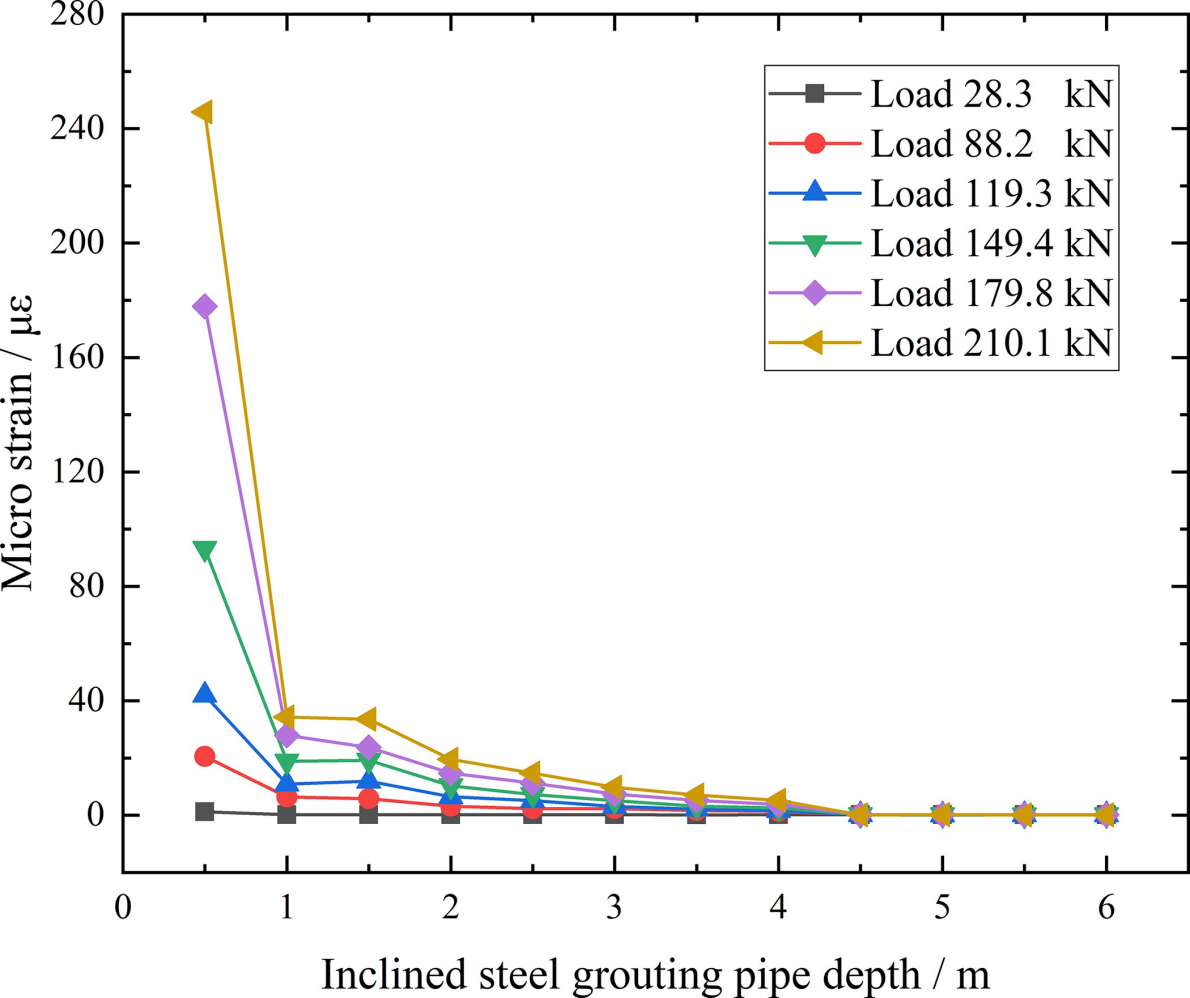
Micro strain corresponding anchorage section depth curve of inclined steel grouting pipe of number 12–3.

It has been proved that anchorage lengths have less influence to bearing capacity of inclined steel grouting pipes in loess embankment slope, which is due to steel pipes in anchorage section have reached its yield point during pull-out tests. Therefore, it is interesting to know how far load could be transferred along anchorage section, which is defined as effective anchorage length. To measure strains along anchorage section of inclined steel grouting pipes in the process of pull-out tests, a series of strain gauges are equipped on its outer surface. Under the action of loading to inclined steel grouting pipes, if the load can transfer to a certain depth along anchorage section, the strain gauge at the same depth will monitor the corresponding strain change. Since micro strain values from readings of VW-100 vibrating string reading meter are also slightly affected by surrounding vibration, micro strain values less than 1 με could be regarded as unchanged. Inclined steel grouting pipes labeled as number 6–3, 9–3, and 12–3 are selected for this test. Strain gauges are equipped along anchorage section with the distance interval of 0.5 m. Micro strain corresponding anchorage section depth curves of inclined steel grouting pipes of number 6–3, 9–3, and 12–3 are displayed in Figs 24–26. The horizontal ordinates of Figs 24–26 has different origins, which correspond to the distances between the top of the inclined steel grouting pipes and the beginning of the anchorage section. These distances are 6 m, 3 m, and 0 m, respectively, and they reflect the free section lengths of the pipes. Specifically, the pipe in Fig 24 has a free section length of 6 m and an anchorage section length of 6 m. The pipe in Fig 25 has a free section length of 3 m and an anchorage section length of 9 m. The pipe in Fig 26 has a free section length of 0 m and an anchorage section length of 12 m.

From the testing result of Fig 24, as for inclined steel grouting pipe labeled as number 6–3, no strain change along anchorage section is monitored when applying load until 120.4 kN. Only strain gauges along anchorage section at first 1 m have strain change when applying loading of 149.8 kN. With the loading and unloading going on, strain gauges along anchorage section at first 3 m have strain change when applying loading of 180.2 kN. When increasing loading to 242.4 kN, results still indicate that strain gauges along anchorage section at first 3 m have strain change. Therefore, load can only transfer to first 3 m of anchorage section in this testing scenario. Thus, the effective anchorage length of inclined steel grouting pipe with anchorage length of 6 m is 3 m.

From the testing result of Fig 25, as for inclined steel grouting pipe labeled as number 9–3, only strain gauges along anchorage section at first 2 m have strain change when applying loading of 30.3 kN. With the loading and unloading going on, strain gauges along anchorage section at first 3.5 m have strain change when applying loading of 88.2 kN. When increasing loading to 206.6 kN, results still indicate that strain gauges along anchorage section at first 3.5 m have strain change. Therefore, load can only transfer to first 3.5 m of anchorage section in this testing scenario. Thus, the effective anchorage length of inclined steel grouting pipe with anchorage length of 9 m is 3.5 m.

From the testing result of Fig 26, as for inclined steel grouting pipe labeled as number 12–3, only strain gauges along anchorage section at first 0.5 m have strain change when applying loading of 28.3 kN. With the loading and unloading going on, strain gauges along anchorage section at first 4 m have strain change when applying loading of 88.2 kN. When increasing loading to 210.1 kN, results still indicate that strain gauges along anchorage section at first 4 m have strain change. Therefore, load can only transfer to the first 4 m of anchorage section in this testing scenario. Thus, the effective anchorage length of inclined steel grouting pipe with anchorage length of 12 m is 4 m.

Compare the effective anchorage lengths of inclined steel grouting pipes with different anchorage lengths. The effective anchorage length of inclined steel grouting pipe with anchorage length of 6 m is 3 m, and the ratio of effective anchorage length to total anchorage length is 0.50. The effective anchorage length of inclined steel grouting pipe with anchorage length of 9 m is 3.5 m, and the ratio of effective anchorage length to total anchorage length is 0.39. The effective anchorage length of inclined steel grouting pipe with anchorage length of 12 m is 4 m, and the ratio of effective anchorage length to total anchorage length is 0.33. It could be concluded that with the increase of anchorage length, effective anchorage length of inclined steel grouting pipe in loess embankment slope will be slightly increased, while the ratio of effective anchorage length to total anchorage length will be decreased.

## Discussion

This paper has presented an experimental study to investigate the bearing capacity and effective anchorage length of inclined steel grouting pipes with different anchorage lengths. By conducting a series of loading and unloading pull-out tests, bearing capacity and effective anchorage length of inclined steel grouting pipes with anchorage lengths of 6 m, 9 m, and 12 m are investigated. In this section, firstly bearing capacity and modulus of inclined steel grouting pipes with anchorage lengths of 6 m, 9 m, and 12 m are compared and analyzed. Secondly, cohesive strength between cement grouting and soil stratum of inclined steel grouting pipe is compared with that of rock bolt. Thirdly, the embankment slope reinforcement principle of ‘Slope drainage + Properties improvement + Force adjustment’ is proposed.

### Comparison of bearing capacity and modulus of inclined steel grouting pipes with different anchorage lengths

**Fig 27:**
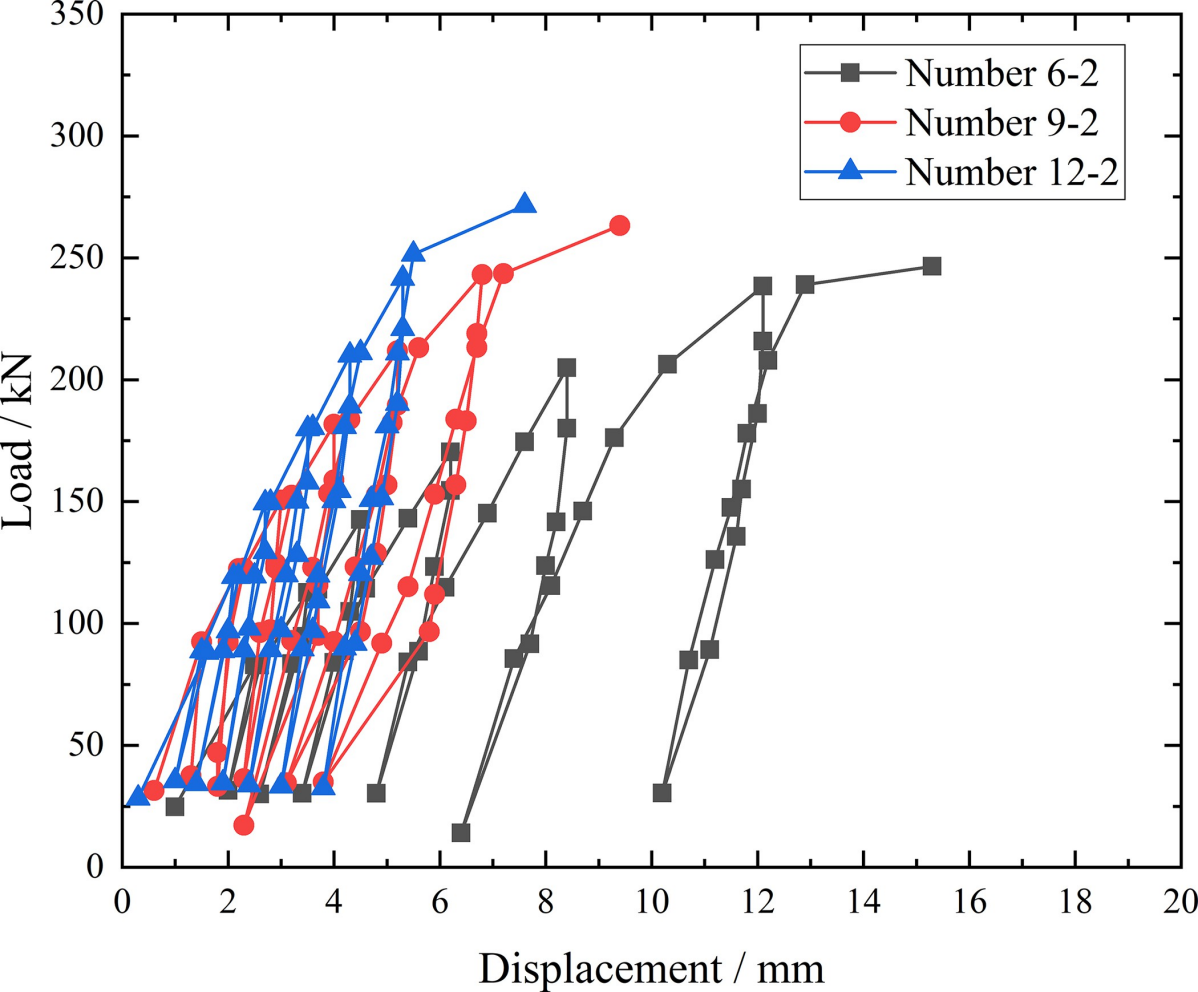
Pull-out test load-displacement curves of inclined steel grouting pipes of number 6–2, 9–2, and 12–2.

From the testing result of Fig 27, by plotting load-displacement curves of inclined steel grouting pipes of number 6–2, 9–2, and 12–2 in the same graph, it is clear that anchorage lengths have less influence to bearing capacity of inclined steel grouting pipes in loess embankment slope since the bearing capacities in three tests are almost at the same level. However, modulus of load-displacement curves of inclined steel grouting pipes of number 6–2, 9–2, and 12–2 are different.

**Fig 28:**
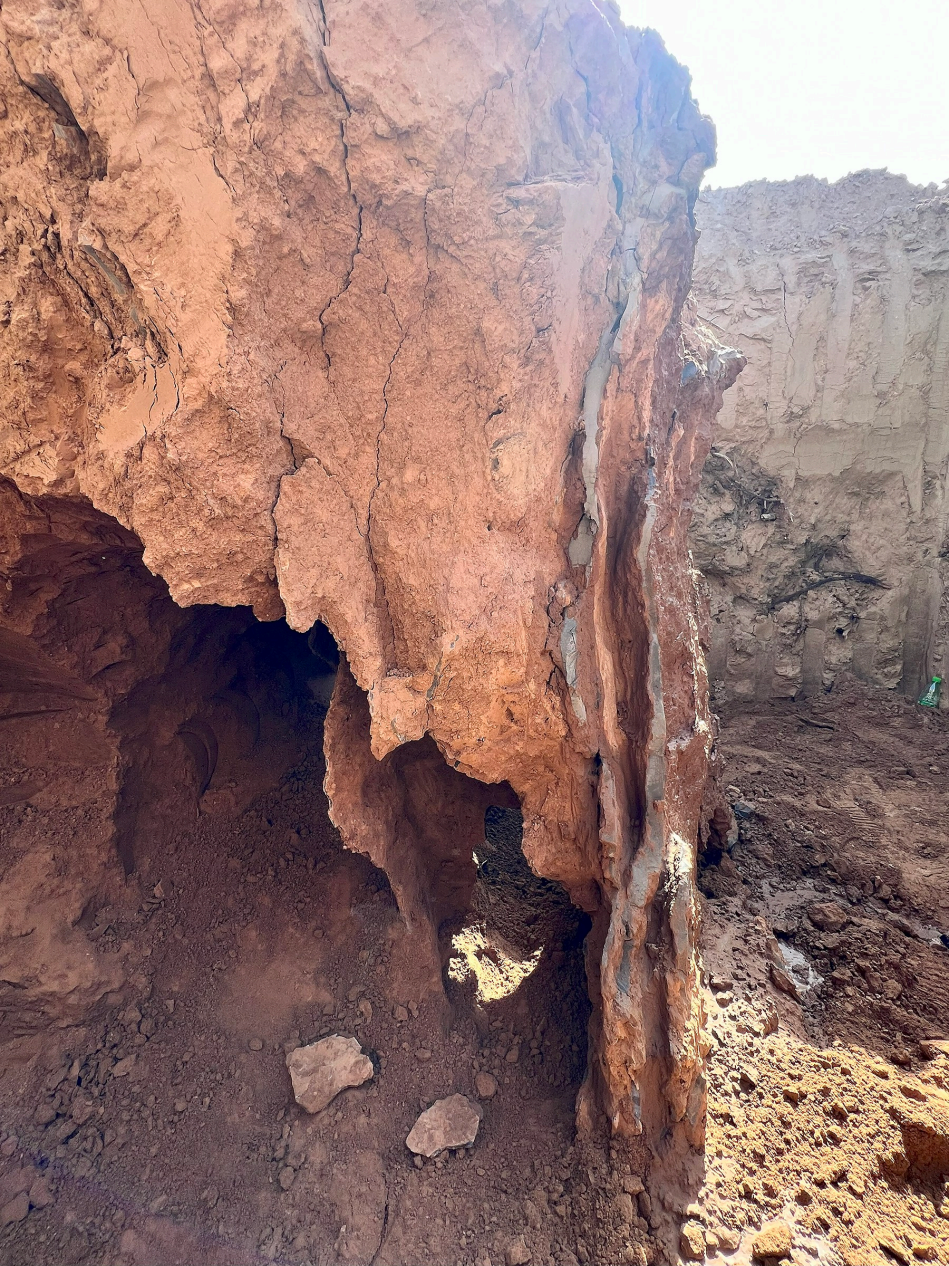
Overall view of the slurry.

**Fig 29:**
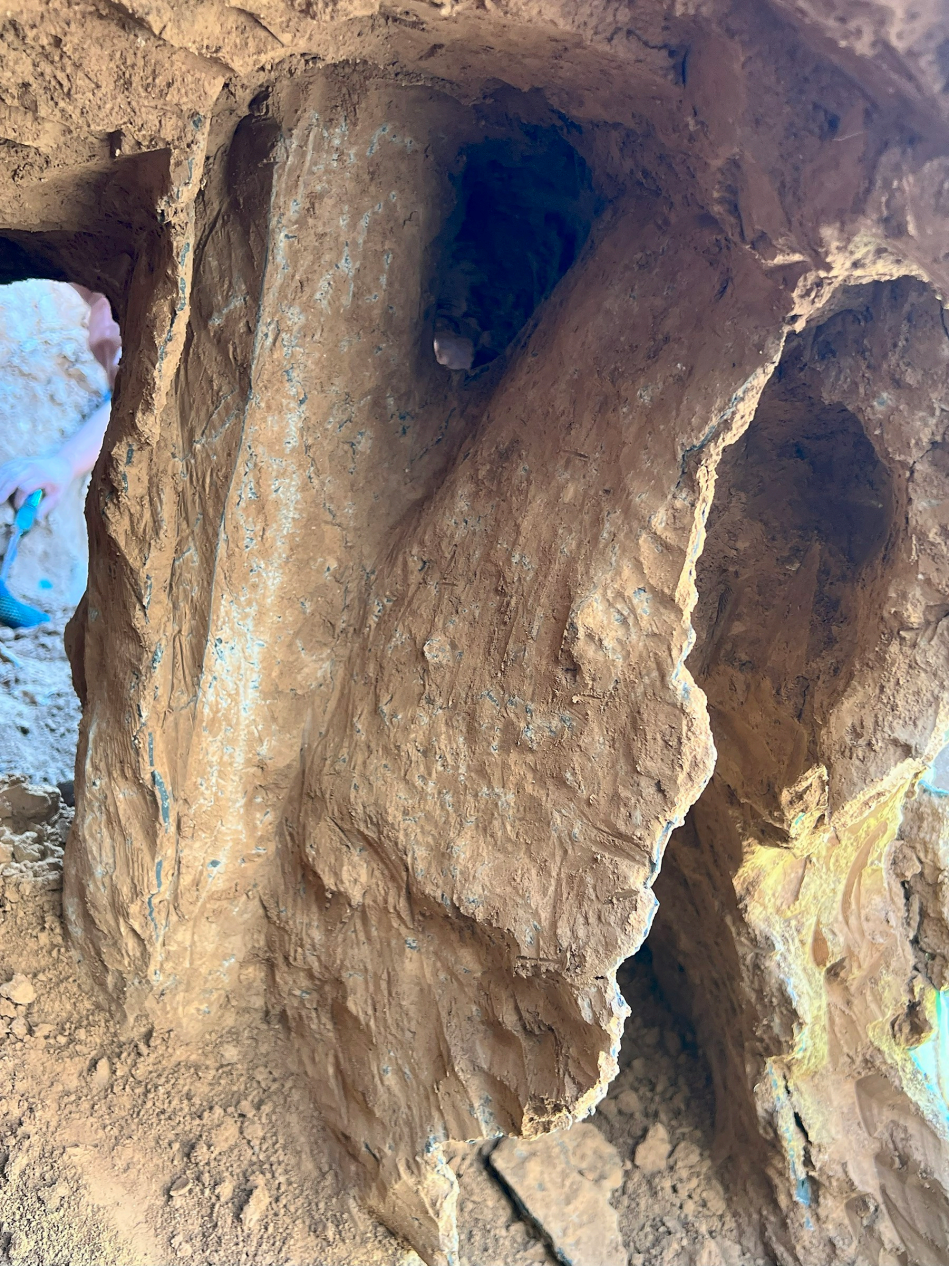
Close-up view of the slurry.

The bearing capacity of inclined steel grouting pipe is dominated by the minimum value of three factors that are tensile strength of the steel pipe, cohesive strength at the interface between cement grouting and soil, and cohesive strength at the interface between steel pipe and cement grouting. The bearing capacity of regular structures such as rock bolts or soil nails is typically dominated by cohesive strength at the interface between cement grouting and soil, which is due to the fact that the cohesive strength at the interface between cement grouting and soil of rock bolts or soil nails is relatively low. When subjecting pull-out loads on rock bolts or soil nails, the interface between cement grouting and soil tends to fail while the loads have not exceeded the tensile strength of the steel pipe. However, the cohesive strength at the interface between cement grouting and soil of inclined steel grouting pipe is relatively high. When subjected to loads of around 250 kN, steel pipes tend to experience tensile failure while the interface between cement grouting and soil does not experience shear failure. This is because splitting grouting significantly enhances the bonding between the cement grouting and the soil. Additionally, excavation prospecting around inclined steel grouting pipes has revealed that cement slurry veins are intricately filled around the steel pipe, providing evidence of the effectiveness of splitting grouting (Figs 28 and 29). Therefore, the bearing capacity of inclined steel grouting pipes is ultimately determined by the tensile strength of the steel pipe. Since inclined steel grouting pipes with anchorage lengths of 6 m, 9 m, or 12 m use the same type of steel pipes, their bearing capacities are expected to be at the same level.

The modulus of load-displacement curve of inclined steel grouting pipe of number 6–2 is lowest, while the modulus of load-displacement curve of inclined steel grouting pipe of number 12–2 is largest. This phenomenon makes sense since inclined steel grouting pipe with anchorage length of 12 m has more splitting grouting segment, hence could provide more friction during pull-out test. Thus, inclined steel grouting pipe with anchorage length of 12 m induces less displacement compared with those with anchorage length of 6 m or 9 m when applying loads at same level. Therefore, inclined steel grouting pipe with anchorage length of 12 m has the largest modulus.

### Comparison of cohesive strength between cement grouting and soil stratum of inclined steel grouting pipe with that of rock bolt

Two interfaces of inclined steel grouting pipe might produce potential shear failure during pull-out test, which are the interface between steel pipe and cement grouting as well as the interface between cement grouting and soil stratum. By conducting plenty of indoor pull-out tests, it turned out that the shear failure occurred at the interface between cement grouting and soil stratum since cohesive strength at this interface is lower [24–27]. Thus, the cohesive strength at the interface between cement grouting and soil stratum is critical for design work.

As for the commonly used structure of rock bolt, its cohesive strength at the interface between cement grouting and soil stratum has been concluded the standard values by previous research [28–31]. According to Chinese protocol ‘Technical specification for geotechnical rock bolt’, cohesive strength at the interface between cement grouting and soil stratum of rock bolt in loess silty clay is 30~80 kPa corresponding to consistency of loess silty clay from soft to stiff. Its average value 55 kPa could be regarded as the average cohesive strength. Cohesive strength at the interface between cement grouting and soil stratum of rock bolt in loess silt is 70~125 kPa. Its average value 97.5 kPa could be regarded as the average cohesive strength.

As for inclined steel grouting pipe, its minimum average cohesive strength along effective anchorage length section at the interface between cement grouting and soil stratum could be calculated by Eq 1.


$$\tau_{u}=\frac{F_{u}}{\pi dl_{e}}$$ (1)

In Eq 1, *τ*_*u*_ is the minimum average cohesive strength along effective anchorage length section. *F*_*u*_ is the bearing capacity of inclined steel grouting pipe. *d* is the diameter of steel pipe. *l*_*e*_ is the effective anchorage length of inclined steel grouting pipe.

As for inclined steel grouting pipe labeled as number 6–2, its bearing capacity is 239 kN, and its effective anchorage length is 3 m. The diameter of the steel pipe is 0.06 m. Thus, its minimum average cohesive strength along effective anchorage length section could be calculated as 422 kPa. As for inclined steel grouting pipe labeled as number 9–2, its bearing capacity is 244 kN, and its effective anchorage length is 3.5 m. The diameter of the steel pipe is 0.06 m. Thus, its minimum average cohesive strength along effective anchorage length section could be calculated as 370 kPa. As for inclined steel grouting pipe labeled as number 12–2, its bearing capacity is 251 kN, and its effective anchorage length is 4 m. The diameter of the steel pipe is 0.06 m. Thus, its minimum average cohesive strength along effective anchorage length section could be calculated as 333 kPa.

By comparing inclined steel grouting pipe minimum average cohesive strength along effective anchorage length section at the interface between cement grouting and soil stratum with cohesive strength at the interface between cement grouting and soil stratum of rock bolt, the value of cohesive strength enhances at least three times. Therefore, compared with rock bolt, inclined steel grouting pipe can largely improve the mechanical property of loess embankment slope.

### Reinforcement principle

The embankment slope reinforcement principle of ‘Slope drainage + Properties improvement + Force adjustment’ is proposed. Based on plenty of loess embankment slope failure cases, the instability of embankment slopes is related to the erosion of water, the deterioration of the mechanical properties of loess, and the excessive sliding force of slope. Therefore, it is critical to adopt a type of structure that can simultaneously achieve this principle to reinforce loess embankment slope.

Previous slope reinforcement technics may only achieve one purpose of slope drainage, properties improvement, or force adjustment. For instance, drainage ditches can only achieve the purpose of slope drainage. Permeation grouting can only achieve the purpose of loess properties improvement. Anti-sliding piles can only achieve the purpose of force adjustment. However, inclined steel grouting pipe can achieve all the purposes of slope drainage, properties improvement, and force adjustment. By injecting slurry into the interior of slope, the pores inside the loess slope are filled, isolating the flow channels inside the slope, effectively reducing the water erosion to the embankment slope, indirectly achieving the purpose of slope drainage. Besides, by splitting grouting into the loess embankment slope, the loess is compacted and densified, thus its cohesion and internal friction angle are enhanced. Meanwhile, the grout fills the cracks of the loess, comprehensively improving the mechanical properties of the loess embankment slope. Additionally, inclined steel grouting pipe can apply adequate anchorage forces on the embankment slope.

## Conclusions

By investigating bearing capacity and effective anchorage length of inclined steel grouting pipes with anchorage lengths of 6 m, 9 m, and 12 m, the following conclusions are proposed.

1. Anchorage length is not a significant influence factor for bearing capacity of inclined steel grouting pipes in loess embankment slope. The bearing capacity of inclined steel grouting pipes with anchorage length of 6 m or 9 m are almost at the same level, and the bearing capacity of inclined steel grouting pipes with anchorage length of 12 m only enhances about 10 kN.
2. Compared with ordinary grouting pipe, the bearing capacity of inclined steel grouting pipe with anchorage length of 9 m increases 22.6% because of the effect of splitting grouting.
3. Anchorage length is a significant influence factor for modulus of load-displacement curves of inclined steel grouting pipes in loess embankment slope. The longer anchorage section is, the larger modulus will be.
4. With the increase of anchorage length, effective anchorage length of inclined steel grouting pipe in loess embankment slope will be slightly increased, while the ratio of effective anchorage length to total anchorage length will be decreased.
5. Inclined steel grouting pipe average cohesive strength along effective anchorage length section at the interface between cement grouting and soil stratum is more than 300 kPa, which is at least three times compared with that of rock bolt.

## Supporting information

S1 File Data of inclined steel grouting pipe pull-out tests. (XLSX)
